# Supplementary material for: The roles of TGF-β, Wnt, and MAPK signaling pathways in joint lineage specification in vitro and ex vivo
Source: Stem Cell Reports. 2025 Oct 23;20(11):102685. doi: 10.1016/j.stemcr.2025.102685 (PMC12790734; doi:10.1016/j.stemcr.2025.102685)
Supplement: Document S1. Figures S1–S6 [file mmc1.pdf]

Stem Cell Reports, Volume *20*

Supplemental Information

The roles of TGF-β, Wnt, and MAPK signaling pathways in joint lineage specification *in vitro* and *ex vivo*

Suyash Raj, Thomas Cutia, Stefano Menghini, Mireia Alemany-Ribes, Junming Cai, Mariel Young, Sarah K. Jachim, Terence D. Capellini, and April M. Craft

The roles of TGFβ, Wnt, and MAPK signaling pathways in joint lineage specification *in vitro* and *ex vivo*

Suyash Raj[1], Thomas Cutia[1], Stefano Menghini[1], Mireia Alemany-Ribes[1,2], Junming Cai[1], Mariel Young[3], Sarah K. Jachim[1], Terence D. Capellini[3,4], April M. Craft[1,2,5]

Affiliations

[1] Department of Orthopedic Surgery, Boston Children's Hospital, Boston, MA, United States

[2] Department of Orthopedic Surgery, Harvard Medical School, Boston, MA, United States

[3] Human Evolutionary Biology, Harvard University, Cambridge, MA, United States

[4] Broad Institute of MIT and Harvard, Cambridge, MA, United States

[5] Harvard Stem Cell Institute, Cambridge, MA, United States

*corresponding author: April.Craft@childrens.harvard.edu

Supplementary information

Supplemental Figures S1–S6

Supplemental Experimental Procedures

Table S1. Metadata and differentially expressed genes between raw clusters in individual Seurat objects, related to Figure S4.

Table S2. Differentially expressed genes between named clusters in the integrated Seurat object, related to Figure 4 and Figure S5.

Table S3. Differentially expressed genes between the Chondrogenic cluster of TGFβ and TGFβ+IWP2 and differentially expressed genes between the Connective Tissue 2 cluster of T+PD and T+βFGF, related to Figure 5.

Supplemental Figure 1

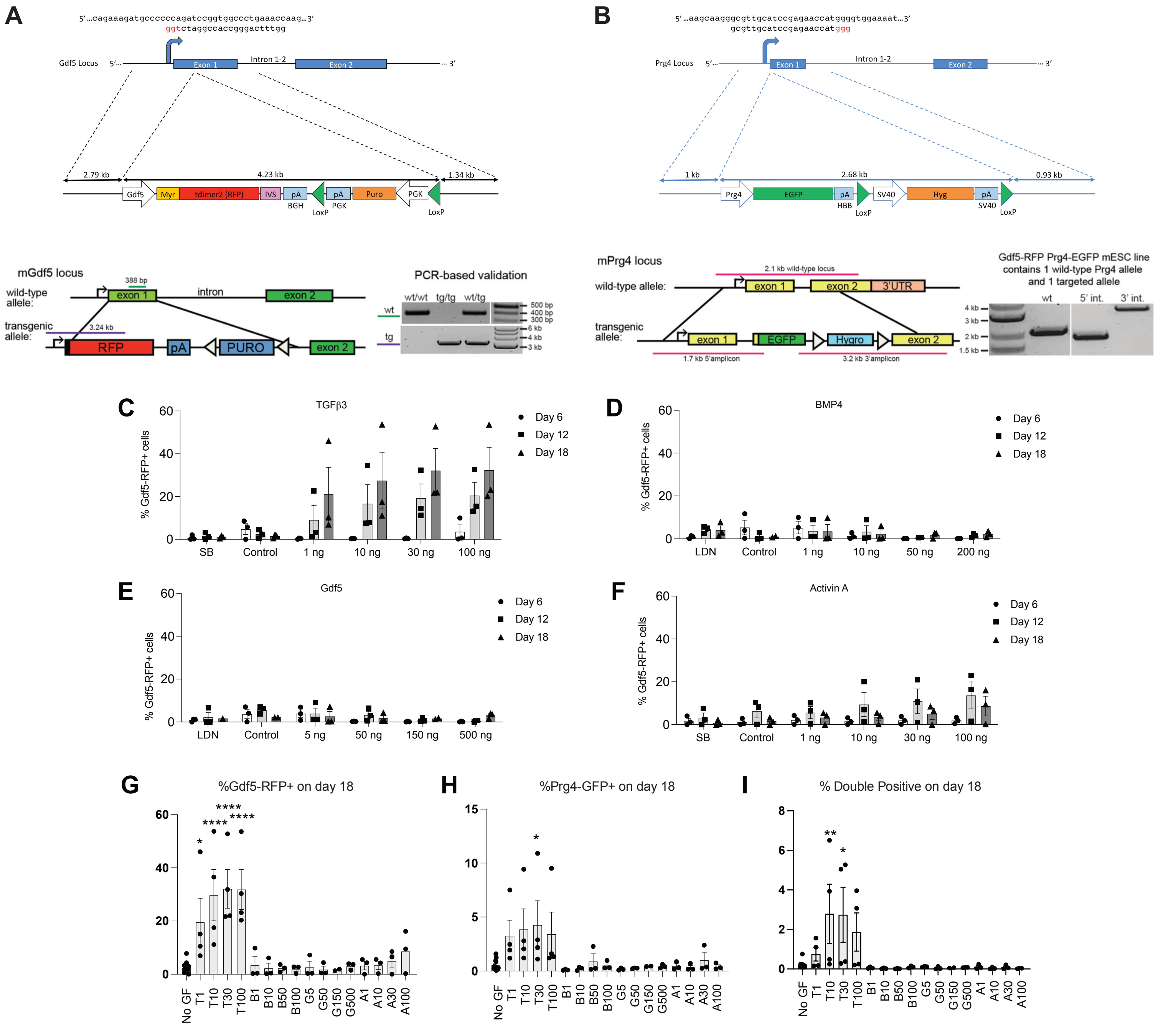

Figure S1. CRISPR-Cas9-mediated generation of the BC29 dual reporter mESC line and activation of the reporters by TGFβ signaling pathway ligands.

(A) Gene targeting and homologous recombination strategy to insert transgene into the Gdf5-RFP locus (top), accompanied by PCR confirmation of recombination (bottom). PCR products were confirmed by sequencing.

(B) Gene targeting and homologous recombination strategy to insert transgene into the Prg4-GFP locus (top), accompanied by PCR confirmation of recombination (bottom). PCR products were confirmed by sequencing.

(C) Percent of Gdf5-RFP expressing cells on days 6, 12 and 18 of micromass culture in the presence or absence (control) of increasing concentrations of TGFβ3 or its antagonist SB431542 (SB), quantified by flow cytometry. Icons represent independent experiments.

(D) Percent of Gdf5-RFP expressing cells on days 6, 12 and 18 of micromass culture in the presence or absence of increasing concentrations of BMP4 or its antagonist LDN-193189 (LDN). Icons represent independent experiments.

(E) Percent of Gdf5-RFP expressing cells on days 6, 12 and 18 of micromass culture in the presence or absence of increasing concentrations of Gdf5 or its antagonist LDN-193189. Icons represent independent experiments.

(F) Percent of Gdf5-RFP expressing cells on days 6, 12 and 18 of micromass culture in the presence or absence of increasing concentrations of Activin A or its antagonist SB431542. Icons represent independent experiments.

(G) Percent of Gdf5-RFP expressing cells on day 18 of micromass culture in the presence or absence of increasing concentrations (numerical values in ng/ml) of TGFβ3 (T), BMP4 (B), Gdf5 (G) or Activin A (A), or no growth factors (No GF). Icons represent independent experiments. Values, mean +/- SEM. ANOVA with Dunnett's multiple comparison correction, *p<0.05, **p<0.01, ***p<0.001, ****p<0.0001 versus no GF.

(H) Percent of Prg4-GFP expressing cells on day 18 of micromass culture in the presence or absence of increasing concentrations (numerical values in ng/ml) of TGFβ3 (T), BMP4 (B), Gdf5 (G) or Activin A (A), or no growth factors (No GF). Icons represent independent experiments. Values, mean +/- SEM. ANOVA with Dunnett's multiple comparison correction, *p<0.05, **p<0.01, ***p<0.001, ****p<0.0001 versus no GF.

(I) Percent of double positive cells on day 18 of micromass culture in the presence or absence of increasing concentrations (numerical values in ng/ml) of TGFβ3 (T), BMP4 (B), Gdf5 (G) or Activin A (A), or no growth factors (No GF). Icons represent independent experiments. Values, mean +/- SEM. ANOVA with Dunnett's multiple comparison correction, *p<0.05, **p<0.01, ***p<0.001, ****p<0.0001 versus no GF.

Supplemental Figure 2

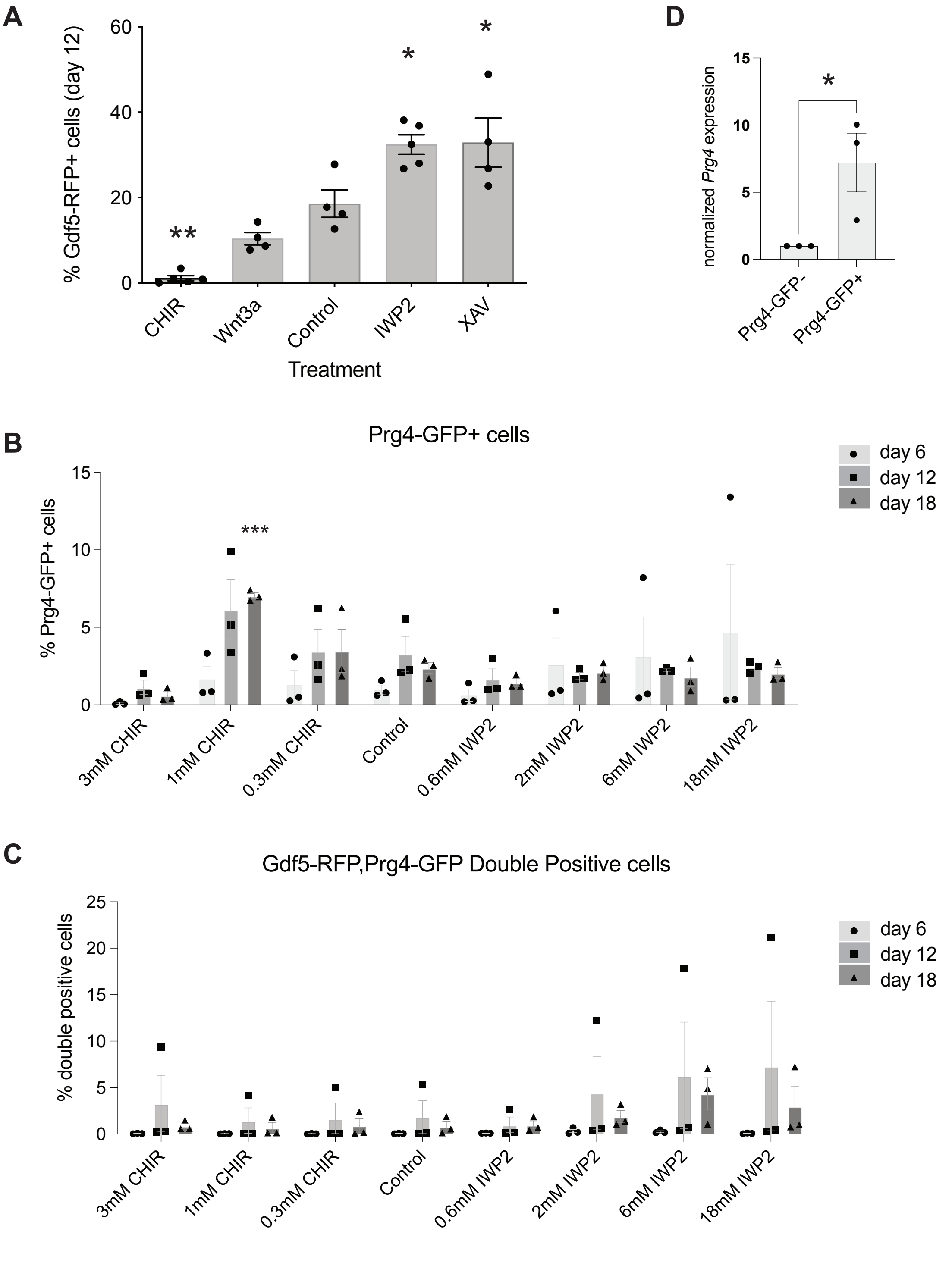

Figure S2. Expression of Gdf5-RFP and Prg4-GFP following modulation of Wnt.
(A) Percentage of Gdf5-RFP expressing cells on day 12 of micromass culture following indicated treatment. n=4-5 independent experiments. Values, mean +/- SEM. One-way ANOVA with Dunnett's multiple comparison correction, *$p<0.05$, **$p<0.01$, versus control (TGFβ3 alone). XAV, XAV939 (2 μM), Wnt3a (100 ng/mL).
(B) Percentage of Prg4-GFP expressing cells on days 6, 12, and 18 of micromass culture in the presence or absence of increasing concentrations of the agonist CHIR99021 or the antagonist IWP2. All treatments include TGFβ3 (10 ng/mL). n=3 independent experiments. Values, mean +/- SEM. One-way ANOVA with Dunnett's multiple comparison correction, ***$p<0.001$, versus control within each timepoint (TGFβ3 alone).
(C) Percentage of Gdf5-RFP;Prg4-GFP (double positive) expressing cells on days 6, 12, and 18 of micromass culture in the presence or absence of increasing concentrations of the agonist CHIR99021 or the antagonist IWP2. n=3 independent experiments, values, mean +/- SEM. One-way ANOVA with Dunnett's multiple comparison correction, all treatments not significant versus control within each timepoint.
(D) Normalized expression of *Prg4* and *Gdf5* mRNA following cell sorting of Prg4-GFP+ and Prg4-GFP+ cells from 3 independent experiments (cultured in the presence of TGFβ3). n=3 independent experiments. Values, mean +/- SEM, Student's t-test, *$p<0.05$.

Supplemental Figure 3

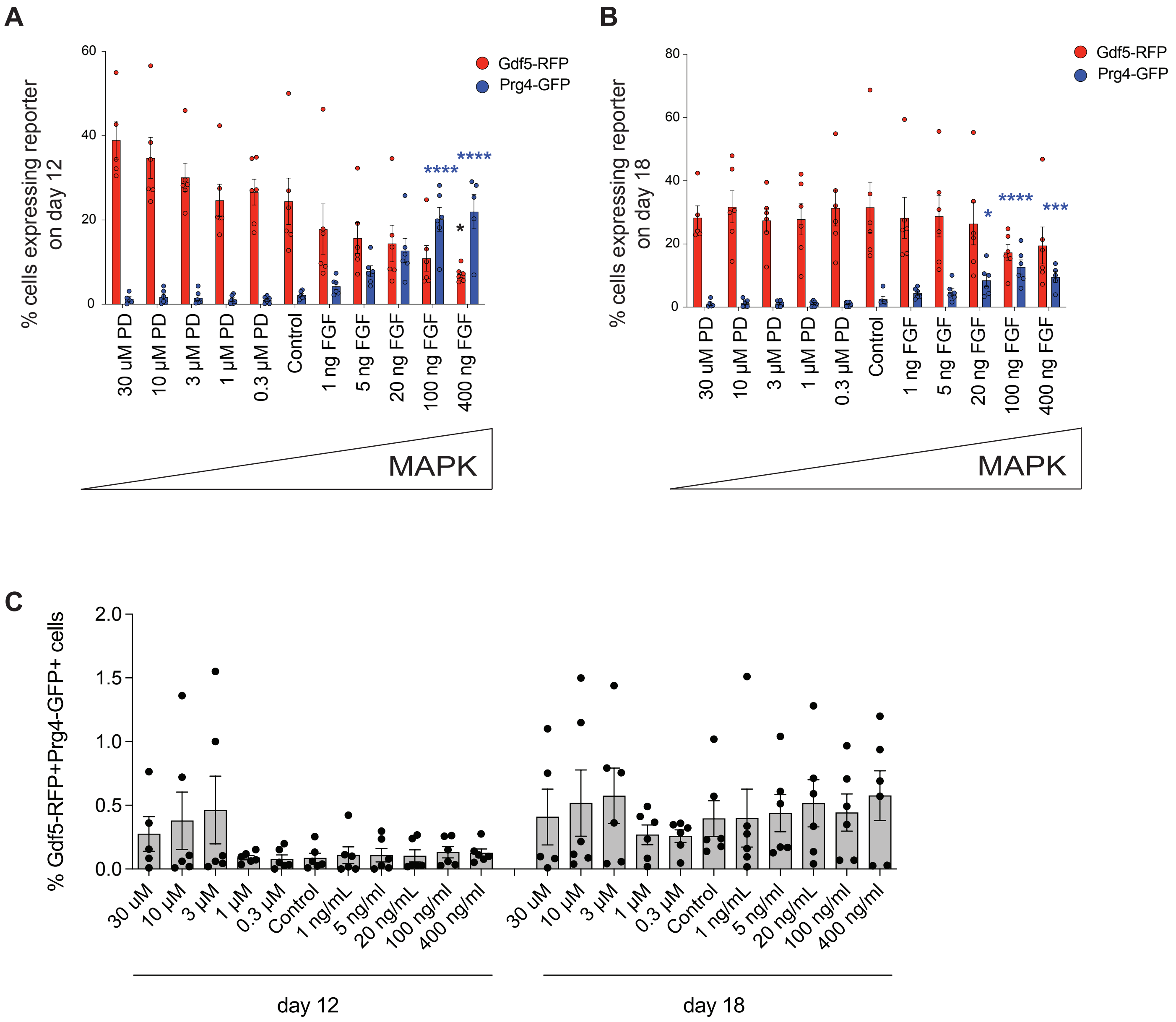

Figure S3. Flow cytometric quantification of Gdf5-RFP and Prg4-GFP following modulation of MAPK signaling.
(A) Percentage of Gdf5-RFP (red bars) or Prg4-GFP (blue bars) expressing cells on day 12 of micromass culture following indicated treatment. Icons represent independent experiments. Values, mean +/- SEM. ANOVA with Dunnett's multiple comparison correction, *p< 0.05, **p< 0.01, ***p<0.001, ****p<0.0001 versus control (TGFβ3 alone).
(B) Percentage of Gdf5-RFP (red bars) or Prg4-GFP (green bars) expressing cells on day 18 of micromass culture following indicated treatment. Icons represent independent experiments. Values, mean +/- SEM. ANOVA with Dunnett's multiple comparison correction, *p< 0.05, **p< 0.01, ***p<0.001, ****p<0.0001 versus control (TGFβ3 alone).
(C) Percentage of Gdf5-RFP;Prg4-GFP-expressing cells (double positive) on day 12 and 18 of micromass culture. Icons represent independent experiments. Values, mean+/- SEM. ANOVA with Dunnett's multiple comparison correction versus control (TGFβ3 alone), results are not significant.

Supplemental Figure 4

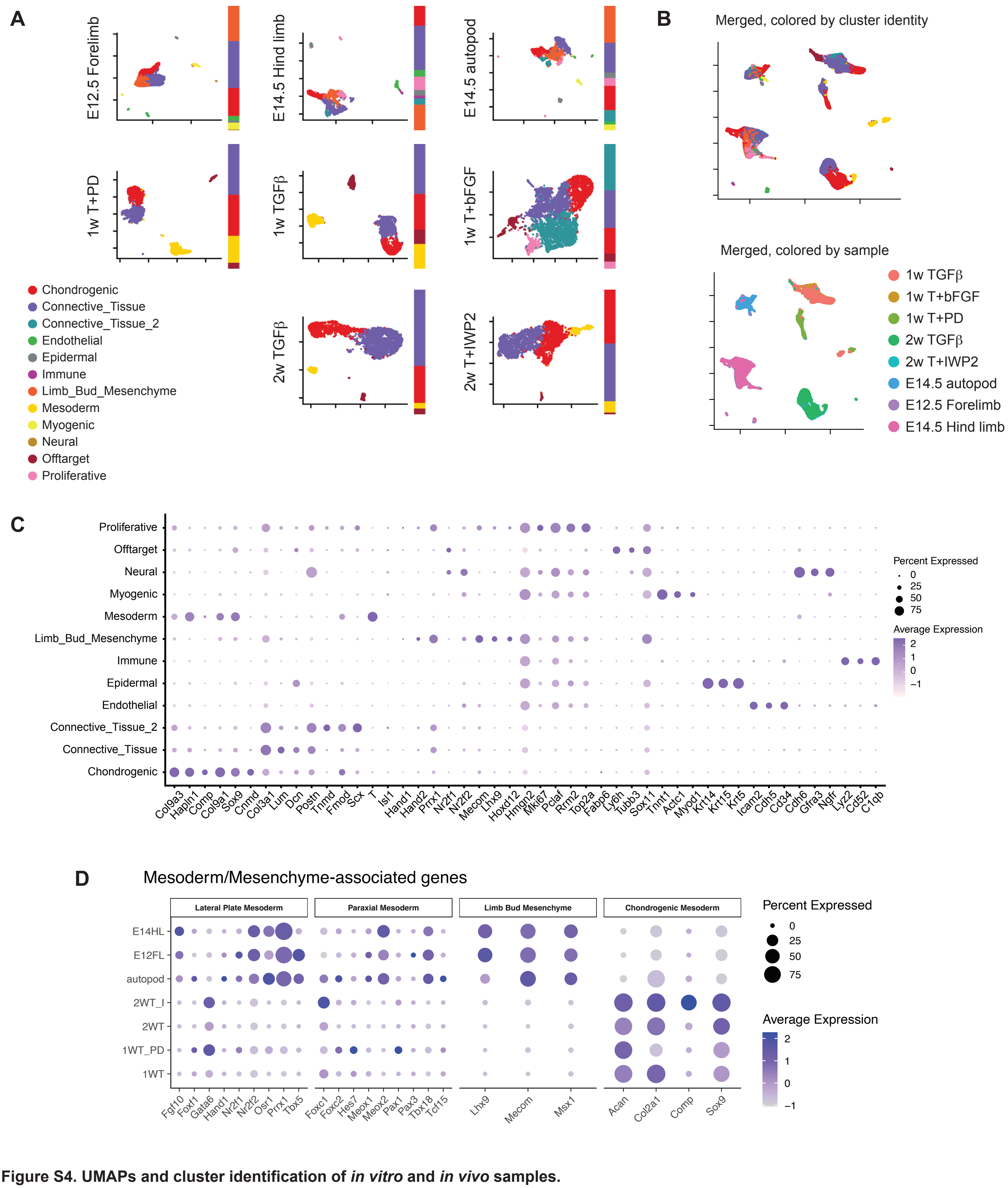

Figure S4. UMAPs and cluster identification of *in vitro* and *in vivo* samples.
(A) UMAPs with cluster identification and proportions in each sample.
(B) Merged RNA object with cluster identification (left) and colored by sample (right).
(C) Semi-quantitative dotplot showing percentage of cells and normalized level of gene expression in each cluster in the merged RNA object (B).
(D) Dotplot showing percentage of cells and normalized level of gene expression in each sample's respective Mesoderm cluster (*in vitro*) or Limb_Bud_Mesenchyme cluster (*in vivo*). Mesoderm clusters (*in vitro*) and Limb_Bud_Mesenchyme clusters (*in vivo*) from the merged RNA object were subset to assess the levels of mesoderm/mesenchyme-associated genes.

Supplemental Figure 5

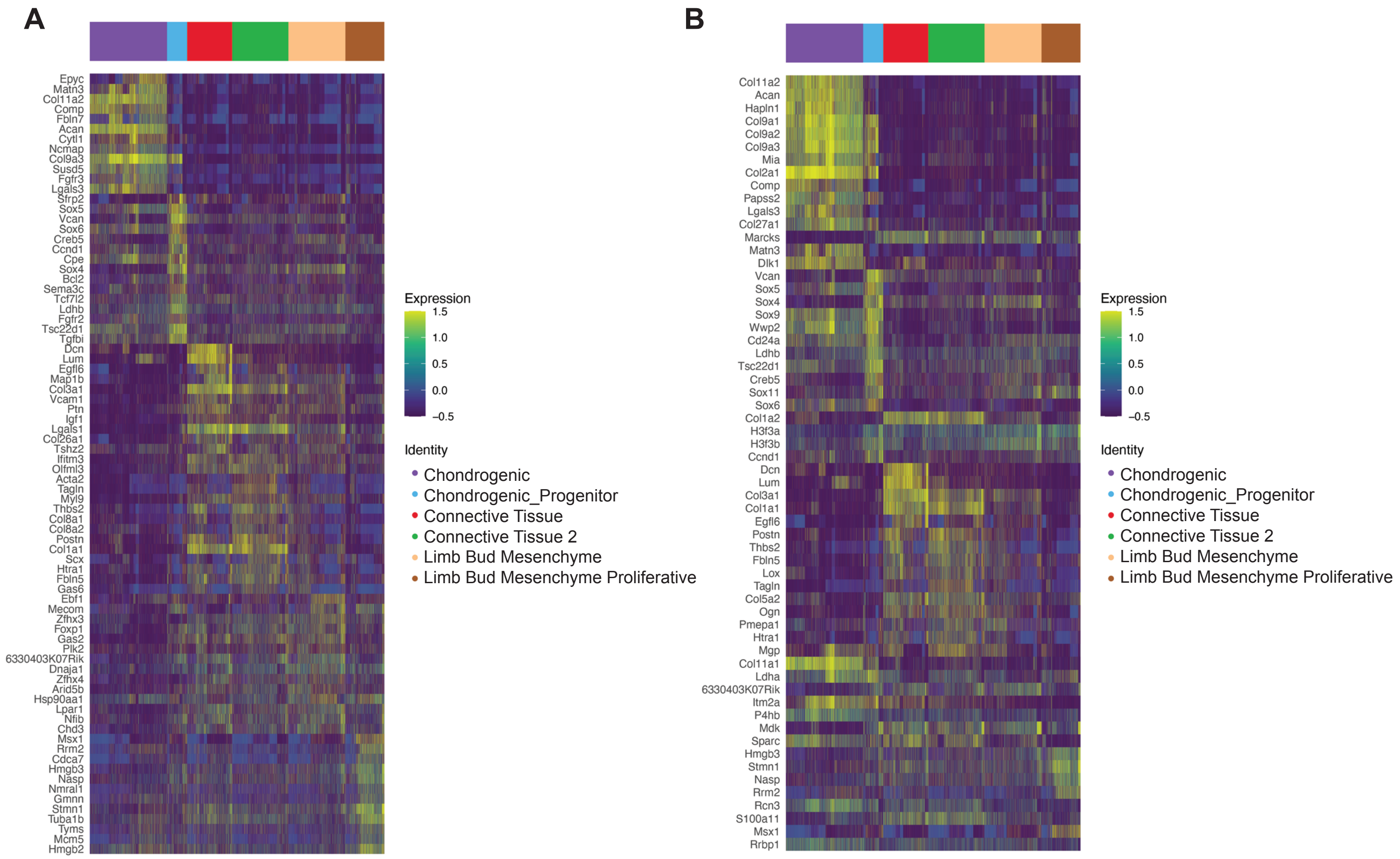

Figure S5. DEGs in integrated object clusters.
(A) Top DEGs per cluster by log(2)FC.
(B) Top DEGs per cluster by p-adj value.

Supplemental Figure 6

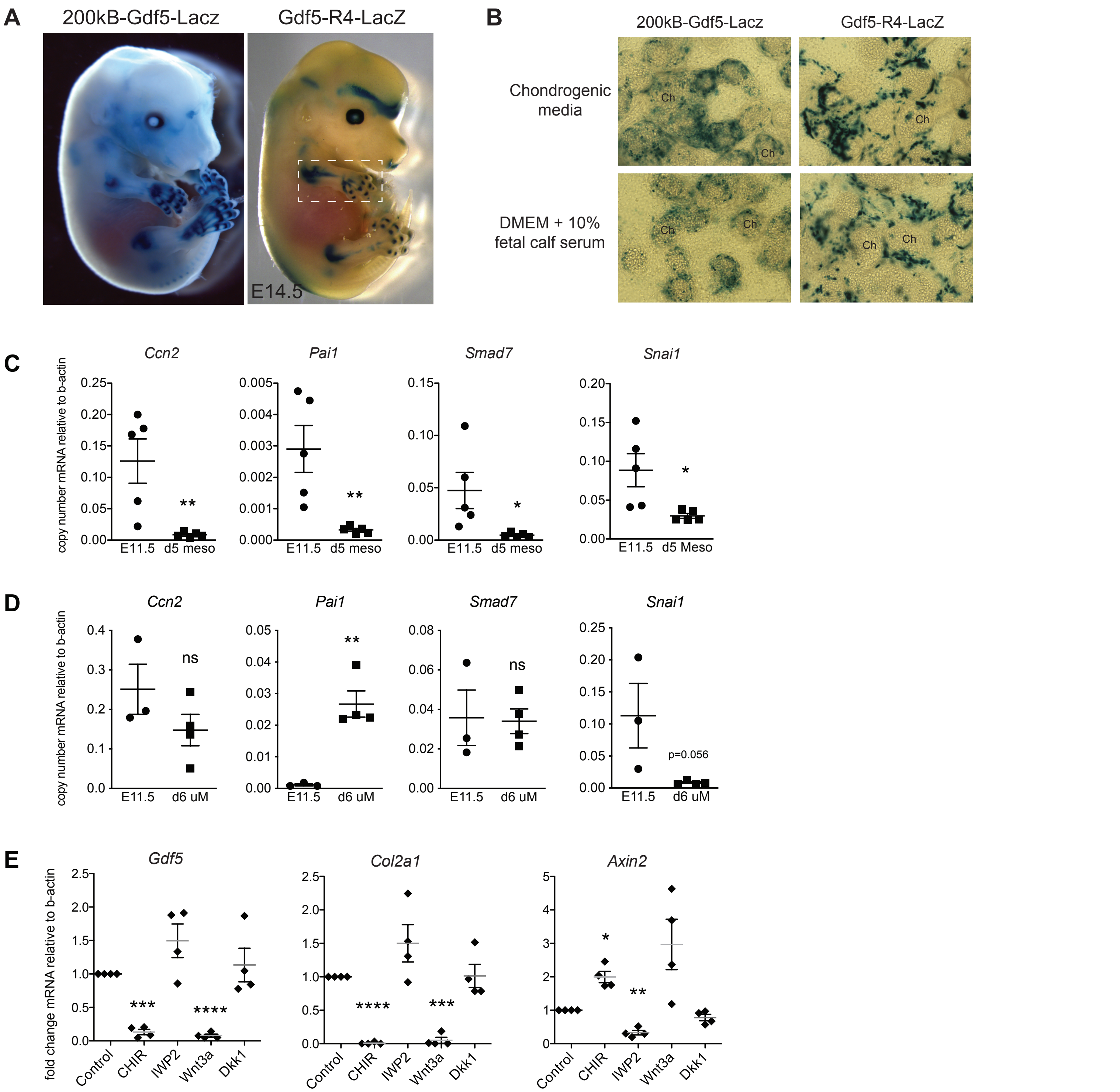

Figure S6. Characterization of mouse embryonic limb bud micromass cultures

(A) X-gal staining of representative mouse embryos on embryonic day 14.5 (E14.5) shows a restricted joint-specific pattern of LacZ-expressing cells in the Gdf5-R4-LacZ transgenic mouse (PHC21) compared to the 200kB-Gdf5-LacZ transgenic mouse, which has a broader expression domain including joint cells and chondrogenic cells.

(B) Localization of LacZ-expressing cells following 8 days of micromass culture in chondrogenic media used in mESC micromass culture (top panels) or traditional limb bud micromass media DMEM + 10% fetal calf serum (bottom panels). Cells from E11.5 limb buds were isolated and plated in micromass culture for 8 days in indicated media. Ch, chondrogenic node. Scale bar, 200 μm.

(C) RT-qPCR of genes associated with TGFβ signaling in pooled limb buds from FVB strain embryos at E11.5 and mESC-derived mesoderm on day 5 of differentiation. n = 5 independent experiments, Values, mean +/- SEM. Student's t-test, *$p<0.05$, **$p<0.01$.

(D) RT-qPCR of genes associated with TGFβ signaling in pooled limb buds from FVB strain embryos at E11.5 (n = 3 independent experiments) and mESC-derived cells on day 6 of micromass culture in the presence of TGFβ3 (n = 4 independent experiments). Values, mean +/- SEM. Student's t-test, **$p<0.01$, ns, not significant.

(E) RT-qPCR quantified expression of *Gdf5, Col2a1* and *Axin2* in 4 day old micromass cultures derived from E11.5 FVB strain mouse embryonic limb buds following treatment with recombinant proteins that modulate canonical Wnt signaling compared to small molecule agonist and antagonist treatments. n=4 independent experiments. ANOVA with Dunnett's multiple comparison correction, *$p<0.05$, **$p<0.01$, ***$p<0.001$, ****$p<0.0001$ vs control.

Supplemental Experimental Procedures

Generation of dual mESC reporter line

The E14 mouse cell line was modified for this study. This is a male embryonic stem cell line that was initially generated from mouse strain 12910la. The coding sequence for myristoylated tdRFP was introduced into the Gdf5 locus and eGFP was introduced into the Prg4 locus of E14 mESCs using CRISPR-Cas9 targeting and homologous recombination (Cong *et al*, 2013) to generate the BC29 dual reporter mESC line (Fig. S1A). The Gdf5-RFP targeting plasmid contained tdRFP and a LoxP-flanked puromycin cassette within a 5' arm homologous to the proximal promoter region and a 3' arm homologous to exon 2 and the 3'UTR. A linear piece of DNA containing the transgenic construct within the 5' homologous region and the 3' homology region was nucleofected into E14 mESCs along with the PX330 plasmid (Addgene #42230) containing a guide RNA. Puromycin resistant clones were screened for homologous recombination by PCR and confirmed by Sanger sequencing. The puromycin resistance cassette was removed by transfection of a Cre-recombinase expression plasmid, selection of puromycin sensitive clones, and the lack of puromycin coding sequence was confirmed in the BC29 clone by Sanger sequencing. The reporter gene eGFP and a hygromycin selection cassette were introduced into the Prg4 locus using CRISPR-Cas9 using homologous recombination and confirmed by Sanger sequencing (Fig. S1B). We note that the untargeted allele of Prg4 is missing one cysteine codon in the signal peptide. The pX330-U6-Chimeric_BB-CBh-hSpCas9 plasmid was a gift from Feng Zhang (Addgene plasmid # 42230; RRID:Addgene_42230).

ESC maintenance and differentiation

Parental E14 and BC29 mESCs were maintained in a modified serum-free (SF), feeder-free culture system as described previously (Craft *et al*, 2013; Gadue *et al*, 2006; Ying *et al*, 2003). Serum-free media was supplemented GSK3β inhibitor CHIR99021 and the MEK inhibitor PD0325901 (i.e., 2i media) (Sim *et al*, 2017). For differentiation, ESCs were dissociated and cultured in suspension in serum-free differentiation medium (SFD) (Gouon-Evans *et al*, 2006) without additional growth factors for 48 hours. Embryoid bodies (EBs) were then dissociated and re-aggregated in SFD with the addition of growth factors or inhibitors as indicated [9 ng/ml activin A (inhibin, beta A), 1 µM CHIR99021, 150 ng/ml noggin or 0.1 µM LDN-193189]. EBs were harvested 28 hours later, the cells were dissociated and re-aggregated at 250,000 cells/ml in 24-well ULA dishes (Costar) or 5% polyheme (Poly 2-Hydroxyethyl methacrylate) (Millipore Sigma) coated 6-well plates for 48 hours in SFD containing 10 ng/ml bFGF (FGF2) and 10 µM Y-27632. Aggregates were harvested on day 5 and dissociated to single-cell suspension.

Three dimensional micromass cultures were generated from mESC-derived mesoderm cells on day 5. Briefly, 250,000 cells were seeded onto Matrigel (Corning)-coated wells of 24-well tissue culture plates (Falcon) in 20 µl of chondrogenic media consisting of high glucose DMEM with 1x ITS supplement (Thermofisher), ascorbic acid (50 µg/ml, Sigma-Aldrich), proline (40 µg/ml, Sigma-Aldrich) and dexamethasone (0.1 µM, Sigma-Aldrich). Chondrogenic media was added to cover the adherent cells after 1 h. Recombinant growth factors and small molecules were purchased from R&D Systems and Sigma-Aldrich.

The day of micromass plating was noted as day 0. Micromass cultures were treated on day 2 and day 4 of micromass culture with indicated factors:

Factor	Concentrations
BMP4	1 – 200 ng/mL
Activin A	1 – 200 ng/mL
Gdf5	1 – 200 ng/mL
TGFβ3	1 – 100 ng/mL
IWP2	0.6 – 18 μM
CHIR99021	0.3 – 3 μM
FGF2 (bFGF	1 – 400 ng/mL
PD0325901	0.3 – 30 μM
SB431542	5.4 μM
LDN-193189	0.1 μM

On day 6, cultures were either (i) maintained in their respective treatments or (ii) cultured in chondrogenic media supplemented with TGFβ3 (10 ng/ml) alone, with media changes every 2-3 days. Micromass cultures were harvested as indicated, primarily at days 6, 12, and 18 of micromass culture for gene expression and flow cytometric analyses. Cells/tissues were maintained in a 5% CO_2 environment for the duration of the culture period.

Mouse embryonic limb bud cultures
Animal studies were performed in compliance of ethical regulations and were approved by Animal Resources at Children's Hospital or Harvard University. Wild type FVB mice, transgenic 200kb BAC Gdf5 LacZ mice, and *PHC21* (*Gdf5-R4-LacZ*) mice (both FVB strain) were used for these studies. Limb buds were micro-dissected from mouse E11.5, E12.5, and E14.5 stage embryos. Cells were isolated from limb buds using 0.2% type I collagenase for up to two hours at 37°C. Cells were then plated in micromass culture as described above or subjected to single cell transcriptomic analyses as described below.

Flow cytometry and cell sorting
EBs generated from mESC differentiation experiments were dissociated by incubation with TrypLE (Invitrogen) and stained with following antibodies: anti-mouse Flk-1-biotin, anti-mouse Pdgfrα (CD140a)-allophycocyanin (APC; clone APA5, eBioscience, San Diego, CA, USA), streptavidin-phycoerythrin (PE) or streptavidin-PE-Cy7 (BD Pharmingen). Antibody stains were performed at 4°C in PBS containing 5% (v/v) fetal calf serum (FCS). Micromass cultures derived from mESCs, embryonic limb buds, and limb bud micromass cultures were dissociated using 0.2% type I collagenase. Limb bud-derived cells expressing beta-galactosidase (*Gdf5-R4-LacZ* transgene) were detected using the fluorescein di-V-galactoside (FDG) reagent within the FluoReporter LacZ flow cytometry kit (F1930, Thermofisher) following manufacturer's instructions. Cells were analyzed using a BD FACS Fortessa (Becton Dickinson). Cell sorting was performed using a BD Melody (Becton Dickinson). Dead

cells staining positively with Draq7 (D15105, ThermoFisher) were excluded from analyses. Analysis was performed using FlowJo (Tree Star). Mean, standard errors and all statistical tests were calculated in Prism (Graphpad).

Reverse Transcription Quantitative PCR (RT-qPCR) Quantitative real-time PCR
Total RNA was extracted using the MagMAX mirVana Total RNA kit (Applied Biosystems). RNA (0.1-1 μg) was reverse transcribed with Superscript IV VILO reverse transcriptase (Invitrogen) and treated with ezDNase enzyme (Invitrogen). RT-qPCR was performed on a ViiA 7 Real-Time PCR System with OptiFlex Optics System (Applied Biosystems) using PowerUp SYBR Green PCR kit (Applied Biosystems). Genomic DNA standards were used to evaluate the efficiency of the PCR and calculate the copy number of each gene relative to the expression of *β-actin*. Primers used are listed in the table below. Biological replicates/independent experiments are indicated in figure and figure legends. Mean, standard errors and all statistical tests were calculated in Prism (Graphpad).

Primers used for quantitative real-time PCR

GENE	Forward (5'-3')	Reverse (5'-3')
Gdf5	ACCACGCAGTCATTCAGACCCTAA	TGTTGGCAGAGTCGATGAAGAGGA
Prg4	AGCCAATGAAGAAGTGCACAGGGA	AGGTGTGTGTCTGGAAAGGTCCAA
Col2a1	CCAAACACTTTCCAACCGCAGTCA	AGTCTGCCCAGTTCAGGTCTCTTA
Snai1	GTCTCAGAAGGGACCATGAATAA	ATAGTTCTGGGAGACACATTGG
Ccn2 (Ctgf)	CAAATGCTGTGCAGGTGATAAA	CCTGAGCCAGCCATTTCTTA
Pai1	GGGCACAACACTTTCATTCAG	ACAGTGGACCTTGAGATAGGA
Scx	AAGTTGAGCAAAGACCGTGAC	AGTGGCATCCACCTTCACTA
Axin2	AAGAGAGACCAAGCCGATTGCTGA	AGTCACTAACACGGCGCTACTCAT

Histology, staining and imaging
Micromass cultures derived from limb bud cells were fixed in 4% paraformaldehyde prior to standard X-gal and alcian blue staining protocols. Images were taken using an EVOS Auto FL 2.0 microscope. *In vitro*-derived micromass tissues were cryofrozen in Tissue-Tek optimal cutting temperature (OCT) mounting medium (Sakura, 4583). 5-10 μm sections of micromass tissues derived from mESCs were cut, fixed in 4% paraformaldehyde, and counterstained with DAPI-containing mounting media. Adjacent tissue sections were fixed in 4% paraformaldehyde and stained with Toluidine blue (Electron Microscopy Sciences) to visualize proteoglycans. Live mESC-derived cultures and fluorescent microscopy was performed using a Zeiss LCM800 confocal microscope.

10x chromium scRNA-seq Library Preparation
In vivo and *in vitro* samples were dissociated using collagenase and resuspended at a concentration of one million cells per mL. 10,000 cells from each sample were loaded into the 10x Genomics Chromium Controller.

Libraries were then prepared using Single Cell 3' Library & Gel Bead Kit v2 (10X Genomics). Final cDNA quality was analyzed using a Bioanalyzer (Agilent). Sequencing was performed by the Harvard Medical School Biopolymers Facility using an Illumina NextSeq 500 to approximately 30,000 reads per cell.

scRNA-seq Processing

Sequencing reads were processed by Cell Ranger (version 3.0.0, 10X Genomics). Reads were aligned to the GRCm38(version 100) genome with added RFP and EGFP transgenes. After genome annotation and filtering of empty droplets, 3000 cells at minimum were captured for each sample. The gene barcode matrix was then passed onto Velocyto (version 0.17.0) which produced individual barcode matrices for spliced, unspliced, and ambiguous mRNA (La Manno *et al*, 2018).

Samples were input into Seurat (version 5.2.1). Cells that had less than 500 UMIs or more than 70,000 UMIs were removed. Low quality cells that contained more than 20% mitochondrial DNA were removed from the final dataset. The cell matrix was normalized using SCTransform (Hafemeister & Satija, 2019) which uses Pearson residuals from a regularized negative binomial regression in order to normalize and scale the matrix as well as to regress out the effects driven by cell cycle stages, mitochondrial genes, and technical variation.

Unsupervised clustering and Projection

Uniform Manifold Approximation and Projection (UMAP) was used to perform dimensional reduction and visualization of the clusters (McInnes *et al*, 2018). The python implementation of UMAP-learn was specifically used. The distance metric for UMAP used was Pearson's correlation, which has been shown to outperform Euclidean distance and does not require minimum principal components (Kim *et al*, 2019). Clustering was performed using Leiden algorithm (Traag *et al*, 2019).. The appropriate resolution was identified using the Clustree package (Zappia & Oshlack, 2018). Clusters were identified through Differentially Expressed Genes (DEGs) and gene ontology. The MAST framework was used to identify DEGs for each cluster (Finak *et al*, 2015).

Integration Analysis

To compare *in vitro* and *in vivo* samples, shared cell types were integrated using reciprocal PCA (RPCA). Seurat includes an implementation of RPCA which was used for our analysis (Butler *et al*, 2018). Mouse E14.5 hind limb and E14 hind limb autopod were used as the reference for the integration because they contained the most diverse populations. Feature plots and dot plots were generated using the scaled RNA matrix, and the volcano plot was generated using the enhanced volcano (version 1.24.0) package.

Computational packages and versions used in these analyses

Package	Version	Package	Version
cowplot	1.1.3	MatrixGenerics	1.18.1
EnhancedVolcano	1.24.0	matrixStats	1.5.0
ggrepel	0.9.6	patchwork	1.3.0
enrichR	3.4	SeuratDisk	0.0.0.9021
extrafont	0.19	RColorBrewer	1.1-3
limma	3.62.2	viridis	0.6.5
harmony	1.2.3	viridisLite	0.4.2
Rcpp	1.0.14	writexl	1.5.2
future.apply	1.11.3	readxl	1.4.5
future	1.34.0	lubridate	1.9.4
glmGamPoi	1.18.0	forcats	1.0.0
RcppAnnoy	0.0.22	stringr	1.5.1
plotly	4.10.4	purrr	1.0.4
scales	1.3.0	readr	2.1.5
MAST	1.32.0	tidyr	1.3.1
SingleCellExperiment	1.28.1	tibble	3.2.1
SummarizedExperiment	1.36.0	ggplot2	3.5.1
Biobase	2.66.0	tidyverse	2.0.0
GenomicRanges	1.58.0	dplyr	1.1.4
GenomeInfoDb	1.42.3	reticulate	1.42.0
IRanges	2.40.1	Seurat	5.2.1
S4Vectors	0.44.0	SeuratObject	5.0.2
BiocGenerics	0.52.0	sp	2.2-0
