# Supplementary material for: The roles of TGF-β, Wnt, and MAPK signaling pathways in joint lineage specification in vitro and ex vivo
Source: Stem Cell Reports. 2025 Oct 23;20(11):102685. doi: 10.1016/j.stemcr.2025.102685 (PMC12790734; doi:10.1016/j.stemcr.2025.102685)
Supplement: Document S2. Article plus supplemental information [file mmc5.pdf]

Stem Cell Reports
Article
OPEN ACCESS

The roles of TGF-β, Wnt, and MAPK signaling pathways in joint lineage specification *in vitro* and *ex vivo*

Suyash Raj,[1] Thomas Cutia,[1] Stefano Menghini,[1] Mireia Alemany-Ribes,[1,2] Junming Cai,[1] Mariel Young,[3] Sarah K. Jachim,[1] Terence D. Capellini,[3,4] and April M. Craft[1,2,5,6,*]

[1]Department of Orthopedic Surgery, Boston Children's Hospital, Boston, MA, USA
[2]Department of Orthopedic Surgery, Harvard Medical School, Boston, MA, USA
[3]Human Evolutionary Biology, Harvard University, Cambridge, MA, USA
[4]Broad Institute of MIT and Harvard, Cambridge, MA, USA
[5]Harvard Stem Cell Institute, Cambridge, MA, USA
[6]Lead contact
*Correspondence: april.craft@childrens.harvard.edu
https://doi.org/10.1016/j.stemcr.2025.102685

SUMMARY

The initiation of synovial joint development and subsequent differentiation of progenitor cells toward anatomically and functionally distinct joint tissues are not well understood, despite being highly relevant to joint health and disease. We generated a dual reporter mouse embryonic stem cell (mESC) line to quantify cells expressing growth differentiation factor five (Gdf5), an early marker of joint formation, and Prg4, a lubricating proteoglycan found in joint tissues. Transforming growth factor β (TGF-β) signaling was necessary and sufficient for the induction of Gdf5-RFP and Prg4-GFP. Inhibition of either Wnt or MAPK signaling significantly increased the induction of Gdf5-RFP, while activation of either pathway prohibited this induction. Single cell transcriptomics demonstrated the chondrogenic identity of Gdf5+ cells in *in vitro* cultures and in mouse embryonic limb buds. We validated the roles of these signaling pathways in joint-specific *ex vivo* limb bud cultures. Thus, this *in vitro* model enhances our understanding of joint development and offers new insights into potential therapeutic approaches for joint disorders.

INTRODUCTION

Synovial joints provide smooth articulation crucial for vertebrate mobility and are found in both the axial and appendicular skeleton. During development, joints are initiated within regions of condensing chondrogenic mesenchymal structures through segmentation and emergence of the interzone. Interzone cells differentiate into chondrocytes that deposit extracellular matrix (ECM), resulting in the formation of articular cartilage, which lines the end of long bones providing a lubricating surface and resilience against mechanical load (Pacifici et al., 2006; Koyama et al., 2008). The cells of the interzone can be identified by the expression of *growth differentiation factor five* (*Gdf5*), a member of the transforming growth factor β (TGF-β) superfamily of proteins. As the joints develop, there continues to be an influx of *Gdf5* positive cells that contribute to the formation of the synovial joint (Shwartz et al., 2016). Lineage tracing studies have confirmed that these *Gdf5*-expressing cell populations contribute to articular cartilage, the meniscus, ligaments, and the synovial lining. Cells at the surface of the articular cartilage continue to express *Gdf5* for a short period of time postnatally, while other key proteins such as lubricin (encoded by the gene *Prg4*) persist throughout adulthood (Jay et al., 2000).

While elegant *in vivo* studies provide insights into the signaling pathways that might play a critical role during synovial joint development (Brunet et al., 1998; Hartmann and Tabin, 2001; Guo et al., 2004; Spagnoli et al., 2007), the signals that initiate the emergence of *Gdf5*-expressing cells to begin the process of joint initiation, and whether those signals also induce cells to differentiate into intra-articular *Prg4*-expressing cells, are less understood. TGF-β signaling was initially implicated in many human skeletal patterning disorders, and TGF-β superfamily members have long been known to promote the differentiation of chondrocytes. Spagnoli et al. identified that TGF-β signaling was not only essential to joint development in mice, but one receptor *Tgfbr2* is specifically expressed in developing joints, and its deletion resulted in lack of joint interzone formation (Spagnoli et al., 2007). Consistent with this observation, the directed differentiation method we developed to generate articular cartilage tissues from human pluripotent stem cells (hPSCs), expressing both *GDF5* and *PRG4,* also relies on TGF-β signaling *in vitro* (Craft et al., 2015; Li et al., 2023). In addition to TGF-β, it has been demonstrated in multiple *in vivo* models that joint development is altered in response to modulation of phospho-(p)ERK (Bastow et al., 2005), fibroblast growth factor (FGF) signaling (Wang et al., 2001), SOXC genes (Bhattaram et al., 2014), and Wnt9a/14 (Guo et al., 2004). Given the findings from these *in vivo* studies, we hypothesized that TGF-β, Wnt, and MAPK signaling pathways would influence the initiation and/or specification of joint lineage cells *in vitro*.

Studying joint development *in vivo* remains challenging due its transient nature, the minute quantity of cells

Published by Elsevier Inc. on behalf of International Society for Stem Cell Research.
This is an open access article under the CC BY license (http://creativecommons.org/licenses/by/4.0/).

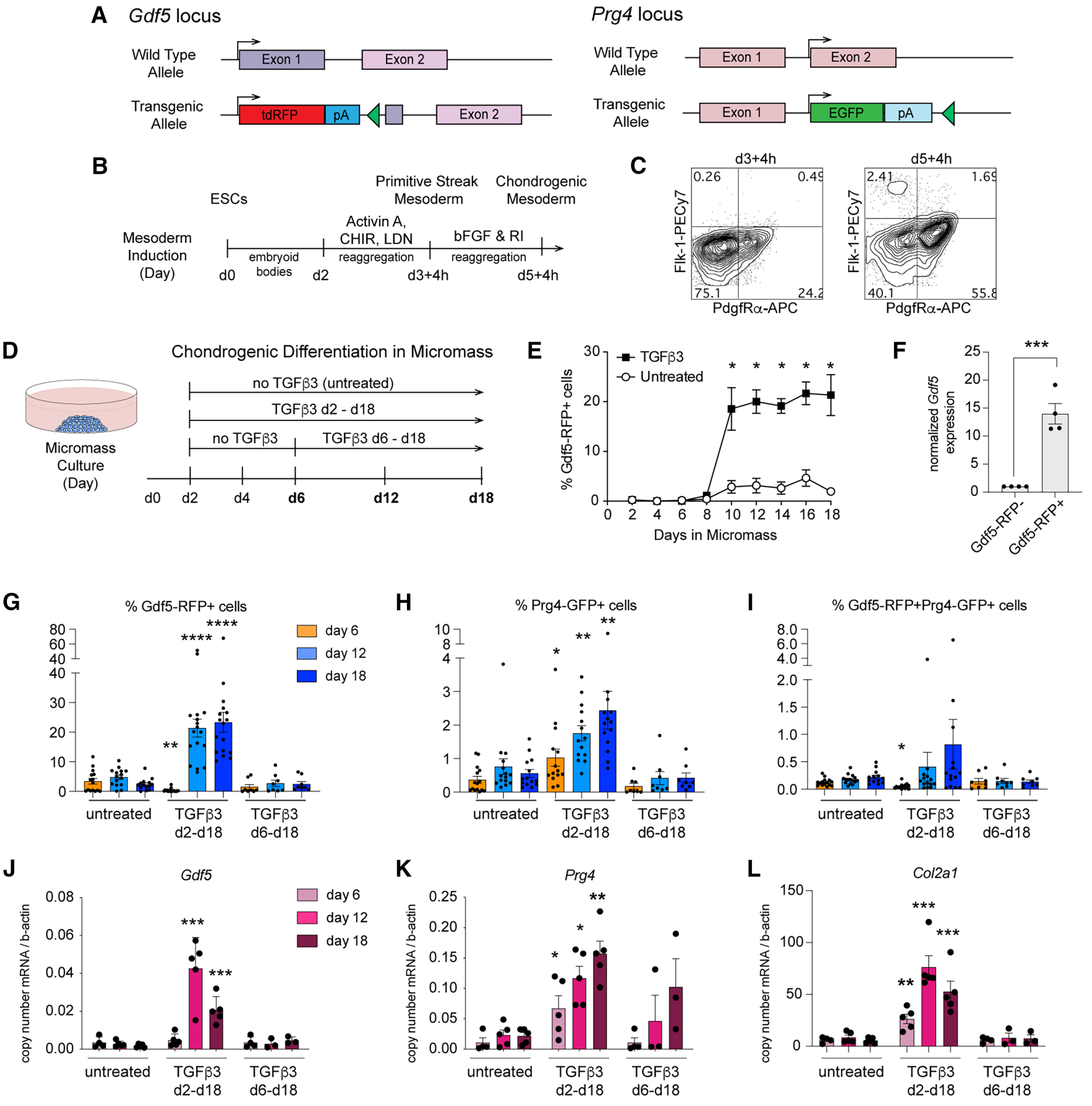

Figure 1. TGF-β signaling is sufficient for induction of Gdf5-RFP expressing cells from mESC-derived mesoderm in micromass culture

(A) CRISPR-Cas9 was used to introduce tdRFP into the first exon of *Gdf5* and EGFP into the first coding exon (exon 2) of *Prg4* in the parental E14 mESC line. The untargeted alleles remain intact.

(B) Methods to induce primitive streak mesoderm and chondrogenic mesoderm from mESCs.

(C) Representative flow cytometry contour plots show expression of Flk-1 and PdgfRα on cells on day 3 and day 5 of differentiation.

(D) Methods to induce chondrogenesis from day 5 mesoderm cells in micromass culture.

(E) Percent of Gdf5-RFP expressing cells during micromass culture in the presence or absence of TGF-β3 (10 ng/mL) from day 2 to day 18, $n = 3$ independent experiments, Student's t test. Values, mean ± SEM. $*p < 0.05$.

(F) Normalized expression of *Gdf5* mRNA following cell sorting of Gdf5-RFP+ and Gdf5-RFP+ cells from 4 independent experiments (cultured in the presence of TGF-β3). Values, mean ± SEM, Student's t test, $***p < 0.001$.

(G) Percentage of Gdf5-RFP+ cells on days 6, 12, and 18 in micromass following indicated treatments (illustrated in D). $n = 8$–15 biological replicates (independent experiments) represented in (G–I); values, mean ± SEM; ANOVA with Dunnett's multiple comparison correction vs. untreated. $*p < 0.05$, $**p < 0.01$, $***p < 0.001$, and $****p < 0.0001$ vs. untreated.

(H) Percentage of Prg4-GFP+ cells on days 6, 12, and 18 in micromass following indicated treatments. Icons represent independent experiments.

(legend continued on next page)

available, and the high costs of generating and maintaining genetically modified animal models. Directed differentiation models using PSCs provide a means to overcome some of the limitations of *in vivo* models, and if directed properly with the correct signals, one could generate an unlimited amount of progenitor cells for downstream studies. While directed differentiation methods that distinguish between articular joint-lining and growth plate cartilage via lateral plate mesoderm remain limited (Smith et al., 2023), we and others have shown articular cartilage potential from PSC-derived paraxial mesoderm populations that are enriched in *GDF5* expression (Craft et al., 2015; Pothiawala et al., 2022), consistent with the finding that the regulatory region of *Gdf5* drives reporter expression in both synovial (e.g., costovertebral and costotransverse) and non-synovial (e.g., intervertebral) joints in the axial skeleton (Chen et al., 2016). These data support the potential use of PSCs for studying the signals that orchestrate joint initiation, and further for generating lineage-restricted intra-articular cells or tissues for replacement or repair.

We aimed to develop new tools and methods to define signals and culture conditions that are sufficient to induce expression of *Gdf5*, marking joint lineage cells, or *Prg4*, expressed by cells on the surfaces of intra-articular tissues. We generated a dual reporter mouse embryonic stem cell (mESC) line with fluorescent proteins expressed by endogenous *Gdf5* and *Prg4* regulatory elements. We found TGF-β signaling to be necessary and sufficient for the induction of *Gdf5* expression. Inhibition of either Wnt or MAPK signals promoted the expression of *Gdf5*, while activation of either pathway diminished it. In contrast, activation of MAPK signaling significantly induced *Prg4* expression. Joint lineage cells induced by combinations of TGF-β, Wnt, and MAPK modulators had similar transcriptomes to chondrogenic and connective tissue lineages isolated from mouse embryonic limb buds. Finally, we demonstrated similar upregulation of joint-enhancer driven *Gdf5* expression upon pathway modulation in *ex vivo* limb bud cultures. These results highlight the utility of this novel reporter mESC line to investigate and quantify the specification of joint lineage cells, shed light on our understanding of this complex developmental process, and support the notion that there are shared mechanisms across joint cell specification in the axial and the appendicular skeleton.

RESULTS

TGF-β signaling is sufficient for the induction of Gdf5-RFP expressing cells from mESC-derived mesoderm

To quantitatively track the emergence of joint lineage cells, fluorescent reporter genes were introduced into the *Gdf5* (tdRFP) and *Prg4* loci (eGFP) using CRISPR-Cas9 methodology, leaving one allele of each intact (Figures 1A and S1). The dual reporter mESC line was specified toward $PdgfRa^{+}Flk\text{-}1^{-}$ primitive streak mesoderm and chondrogenic mesoderm using methods we previously established (Figures 1B and 1C) (Craft et al., 2013). This induction protocol includes BMP inhibitory signals that are consistent with methods developed by us and others to induce paraxial mesoderm from PSCs (Craft et al., 2015; Pothiawala et al., 2022; Tani et al., 2023; Wu et al., 2021). Following mesoderm induction, we introduced the mESC-derived cells to high density micromass culture to induce chondrogenesis, adapting the protocols we established for differentiating hPSCs toward articular cartilage (Figure 1D) (Craft et al., 2015; Richard et al., 2023).

Joint morphogenesis in the limbs *in vivo* and articular chondrogenesis *in vitro* rely on TGF-β, thus, we hypothesized that exogenous TGF-β would be sufficient to induce *Gdf5* and *Prg4* expression. TGF-β3 was introduced to the micromass cultures after a deliberate 48 h grace period to promote cell adherence (Figures 1D and 1E). In the absence of TGF-β3, only 3%–5% of cells express Gdf5-RFP on day 10 and onwards (Figure 1E). In the presence of TGF-β3, Gdf5-RFP was detectable by day 8 in ~2% of cells. By day 10, over 20% of the cells were expressing Gdf5-RFP, which remained relatively stable until day 18. Expression of *Gdf5* mRNA was restricted to Gdf5-RFP+ cells following fluorescence-activated cell sorting (FACS) from TGF-β3-treated micromasses on day 12 (Figure 1F). To determine whether TGF-β is required to induce Gdf5-RFP in the early stage (days 2–6) of micromass culture versus late stage (days 6–18), we delayed the introduction of TGF-β3 in the cultures until day 6, which is prior to the onset of Gdf5-RFP expression in untreated cultures. Flow cytometry on day 6, day 12 (chosen as it captures the initial peak of Gdf5-RFP expression), and day 18 (culture endpoint) showed that TGF-β3 treatment initiating on day 6 did not upregulate the reporter, suggesting that TGF-β signaling was required between days 2 and 6 of micromass culture to sufficiently induce Gdf5-RFP

(I) Percentage of Gdf5-RFP+;Prg4-GFP+ cells (double-positive) on days 6, 12, and 18 in micromass following indicated treatments. Icons represent independent experiments.

(J–L) Expression of indicated genes quantified by RT-qPCR on days 6, 12, and 18 following indicated treatments. $n = 4–6$ independent experiments. Values, mean ± SEM. ANOVA with Dunnett's multiple comparison correction versus untreated. *$p < 0.05$, **$p < 0.01$, and ***$p < 0.001$ vs. untreated. See also Figure S1.

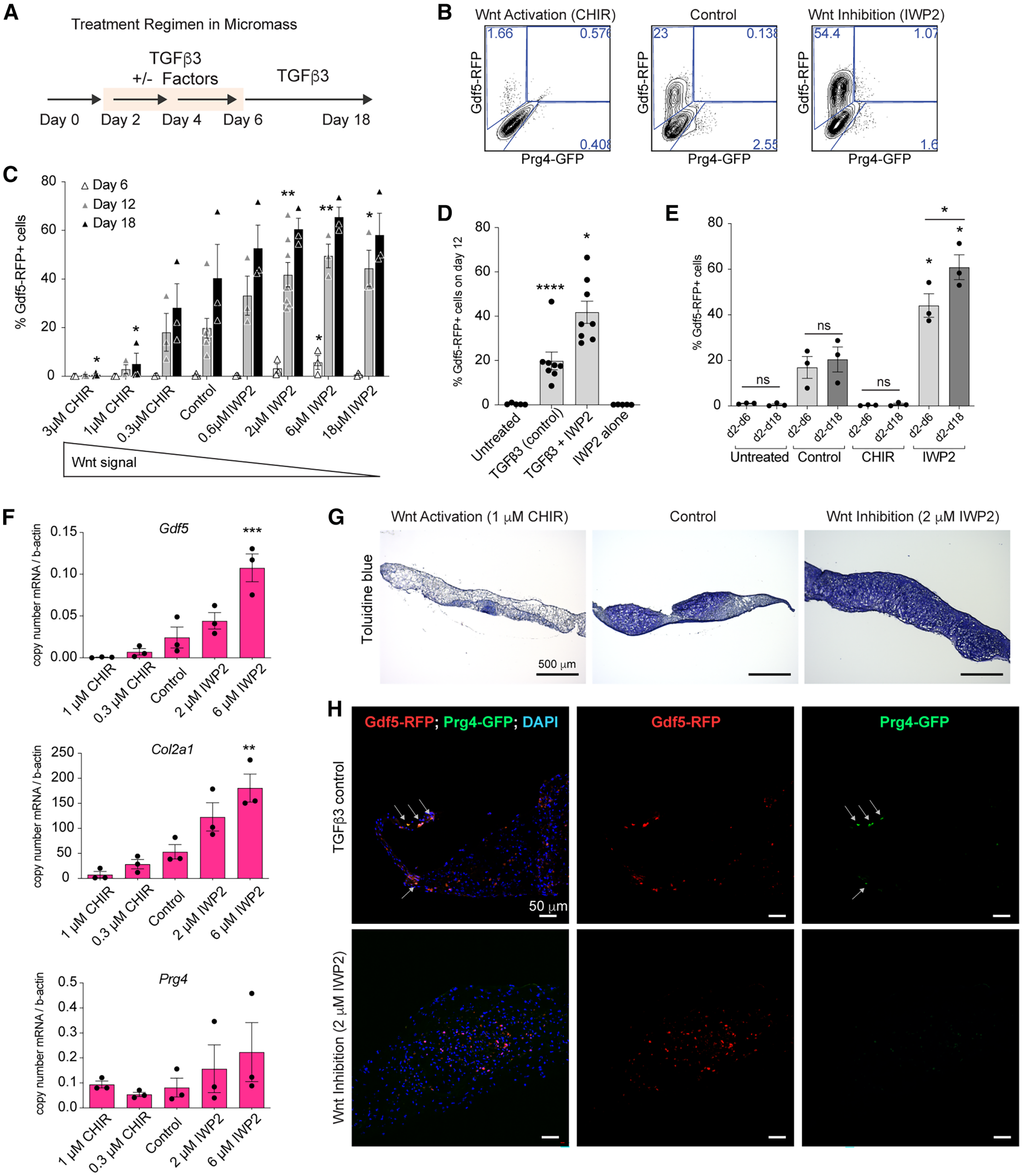

Figure 2. Inhibition of Wnt in the presence of TGF-β significantly increases the percentage of Gdf5-RFP expressing cells and chondrogenic potential in a dose-dependent manner

(A) Treatment scheme of micromass cultures with Wnt pathway agonist (CHIR99021) and antagonist (IWP2).

(B) Representative flow cytometry contour plots show expression of Gdf5-RFP and Prg4-GFP on day 18 of micromass culture.

(C) Percentage of Gdf5-RFP expressing cells on days 6, 12, and 18 of micromass culture in the presence or absence of increasing concentrations of the agonist CHIR99021 or the antagonist IWP2. Values, mean ± SEM. n = 3–8 independent experiments, ANOVA with Dunnett's multiple comparison correction versus control (TGF-β3 alone). *$p < 0.05$ and **$p < 0.01$.

(D) Percentage of Gdf5-RFP expressing cells on day 12 of micromass culture following IWP2 treatment in the presence or absence of TGF-β3 from 8 independent experiments. Values, mean ± SEM. ANOVA with Dunnett's multiple comparison correction versus untreated. *$p < 0.05$ and ****$p < 0.0001$.

(E) Percentage of Gdf5-RFP expressing cells on day 18 of micromass culture following indicated treatment either from day 2–6 (early, light gray bars) or from day 2–18 (continuous, dark gray bars). Icons represent 3 independent experiments. ANOVA with Dunnett's multiple

(legend continued on next page)

(Figure 1G). Prg4-GFP was also induced and was significantly higher in the cultures treated with TGF-β3 from days 2–18 (Figure 1H). Similarly, double-positive cells were in higher abundance in TGF-β3-treated cultures and peaked at about 0.8% on day 18 (Figure 1I), but this was not statistically significant. Expression of *Gdf5* and *Prg4* (Figures 1J and 1K), as well as the cartilage matrix gene *type II collagen* (*Col2a1*, Figure 1L), followed similar patterns, being significantly higher in continuous TGF-β3 treatment cultures. Delayed treatment with TGF-β3 on day 6 was sufficient to observe Prg4-GFP, consistent with previous reports (Zhang et al., 2021; Khalafi et al., 2007) that this gene is responsive to TGF-β signaling.

We next investigated whether other members of the TGF-β superfamily, including Gdf5 itself, could induce Gdf5-RFP (Figures S1C–S1F). We treated micromass cultures with increasing doses of TGF-β3, activin A, BMP4, and Gdf5, as well as with a chemical inhibitor of each ligand (Figures S1C–S1F). TGF-β3 was the only ligand that significantly increased the percentage of Gdf5-RFP+, Prg4-GFP+, and Gdf5-RFP+; Prg4-GFP+ cells compared to untreated cells (Figures S1G–S1I). The induction of Gdf5-RFP was dose dependent with respect to TGF-β3 (Figures S1C and S1G). At 100 ng/mL of activin A, about 10% of cells expressed Gdf5-RFP (Figures S1F and S1G). Neither BMP4 nor Gdf5 were able to induce Gdf5-RFP at any concentration tested (Figures S1D and S1E).

Wnt inhibition in the presence of TGF-β significantly increases the percentage of Gdf5-RFP+ cells

Wnt signaling has been implicated in joint initiation in the limb (Guo et al., 2004), and inhibition of Wnt has been previously shown to improve chondrogenesis from PSCs by minimizing off-target cell types (Wu et al., 2021). We sought to define the role of Wnt in the induction of Gdf5-RFP and Prg4-GFP by using agonists or antagonists between days 2 and 6 of micromass culture (Figure 2A). In the absence of TGF-β3, neither the small molecule Wnt agonist CHIR99061 (CHIR; a GSK3β inhibitor) nor the Wnt antagonist IWP2 (PORCN inhibitor) induced Gdf5-RFP (data not shown, and Figure 2D). In the presence of TGF-β3, addition of CHIR resulted in a loss of Gdf5-RFP+ cells, while inhibition of Wnt with IWP2 significantly increased the percentage of Gdf5-RFP+ cells (up to 2-fold higher) on day 18 (Figures 2B and 2C). By day 12 the induction of Gdf5-RFP became dose dependent on IWP2, where concentrations of 2 μM or higher were significantly higher than TGF-β3 treatment alone (control). Conversely, as CHIR concentrations were increased, the percentage of Gdf5-RFP+ cells was significantly decreased compared to control. Early Wnt inhibition (days 2–6) was sufficient to increase the proportion of Gdf5-RFP+ cells on day 18, while continued IWP2 treatment (days 2–18) led to a slight but significant increase compared to early Wnt inhibition (Figure 2E), suggesting that Wnt inhibition was effective at promoting Gdf5-RFP throughout the differentiation. Similar responses in the percentage of Gdf5-RFP+ cells were observed following the addition of an alternative Wnt antagonist XAV939 or agonist Wnt3a (Figure S2A). Interestingly, the percentage of Prg4-GFP+ cells increased slightly in cultures treated with CHIR on day 18 compared to control, and the percentage of double-positive cells remained unchanged (Figures S2B and S2C).

To identify the fate of these cells, we performed quantitative quantitative reverse-transcription PCR (RT-qPCR) on day 12 of micromass culture (Figure 2F). We observed significant increases in *Gdf5* and *Col2a1* in the presence of IWP2, and increased *Prg4* expression compared to TGF-β3 alone. With CHIR, expression of these genes was either decreased or similar to cultures treated with TGF-β3 alone (Figure 2F). Sulfated glycosaminoglycans (sGAGs), which are abundant in cartilage, were found throughout the TGF-β3+IWP2-treated micromasses using the metachromatic toluidine blue stain (i.e., purple color in Figure 2G). TGF-β-treated tissues also contained areas with abundant sGAGs, while these were absent in CHIR-treated tissues. Gdf5-RFP+ cells were generally found to be evenly distributed within the cartilaginous regions of the TGF-β3-treated and TGF-β3+IWP2-treated tissues (Figure 1H). As expected, very few Prg4-GFP+ cells were observed, but were most abundant at the periphery of the micromass, where they co-expressed Gdf5-RFP (Figure 1H, white arrows in TGF-β3-treated tissue). Prg4-GFP+ cells isolated from micromasses treated with TGF-β3 showed restricted expression of *Prg4* mRNA, but *Gdf5* mRNA was not enriched in either GFP+ or GFP− cells (Figure S2D).

comparison correction for significance versus respective early or continuous control (TGF-β3 alone). Student's *t* test to compare early to continuous within each treatment. $*p < 0.05$.

(F) RT-qPCR of *Gdf5*, *Col2a1*, and *Prg4* on day 12 of micromass culture from 3 independent experiments. Values, mean ± SEM. ANOVA with Dunnett's multiple comparison correction versus control. $**p < 0.01$ and $***p < 0.001$.

(G) Sections of representative micromass tissues were stained with toluidine blue (purple stain indicates sulfated glycosaminoglycans) on day 32. Scale bars, 500 μm.

(H) Gdf5-RFP-expressing and Prg4-GFP-expressing cells in sections of above micromasses were visualized by confocal fluorescence microscopy. Nuclei are stained with DAPI. Scale bars, 50 μm. Cells expressing both Gdf5-RFP and Prg4-GFP are indicated with gray arrows. See also Figure S2.

MAPK inhibition in the presence of TGF-β induces early Gdf5-RFP in the *in vitro* cultures

It has been previously reported that MAPK signaling plays a role in chondrogenic differentiation from bone marrow derived mesenchymal stem cells (Ma et al., 2019; Muddasani et al., 2007), and FGF signaling is active during chondrogenesis in appendicular skeletal elements. Here, we used our *in vitro* model to assess if MAPK signaling plays a role in *Gdf5* induction and joint lineage specification. We introduced an MAPK agonist, basic fibroblast growth factor (FGF-2, bFGF), and an MAPK antagonist (MEK inhibitor PD0325901, referred to as "PD") on day 2 and day 4 of micromass culture in the presence of TGF-β3. Inhibition of MEK with PD resulted in the upregulation of Gdf5-RFP by day 6 in a dose dependent manner (Figures 3A and 3B). Greater than 26% (and up to 55% in individual experiments) of cells were expressing Gdf5-RFP with PD concentrations of 3 μM or higher (Figure 3B), and was significantly higher than untreated cultures on days 6 and 12 (Figures 3E and 3F). PD-treatment alone, however, was not sufficient to induce Gdf5-RFP. Cells expressing both Gdf5-RFP and Prg4-GFP (double-positive) were also significantly higher with PD at 3 μM or higher concentrations, but did not exceed an average of 1.6% (Figure 3B). The introduction of bFGF resulted in significant increases in the percentage of Prg4-GFP+ cells by day 6 in a dose dependent manner, with greater than 16% and up to 48% of the cells expressing Prg4-GFP at concentrations between 20 and 400 ng/mL (Figures 3A and 3B). Prg4-GFP+ cells remained significantly higher in these bFGF-treated cultures compared to control on days 12 and 18 (Figure S3B), while levels of Gdf5-RFP plateaued to those observed with TGF-β3 alone (Figure S3A) and double-positive cells were not significantly different (Figure S3C).

Gdf5-RFP+ cells were found in and between chondrogenic areas of PD-treated micromasses on day 6, while Prg4-GFP+ cells were highly prevalent in the fibroblast-like cells outgrowing from bFGF-treated micromasses, similar to their peripheral location in Figure 1H (Figures 3C and 3D). RT-qPCR for *Gdf5* on day 6 corroborated the increase in Gdf5-RFP+ cells we observed by flow cytometry in the PD-treated cells and confirmed no effect with bFGF (Figure 3G). bFGF caused a significant increased in *Prg4* mRNA compared to control cultures (Figure 3I), but expression was also increased in PD-treated cultures, possibly reflecting the higher percentage of double-positive cells (Figure 3B). However, neither PD nor bFGF resulted in significant increases in *Col2a1* compared to TGF-β3 alone (Figure 3H), rather the highest concentrations of both reduced *Col2a1* expression. From these data, the fate of the PD and bFGF-treated cells remained unclear. As both TGF-β and FGF signaling have been implicated in tendon/ligament lineages (Havis et al., 2016), we quantified the expression of *scleraxis* (*Scx*), a transcription factor expressed in early tendon and ligament fibroblasts (Brent and Tabin, 2004). We observed a significant increase in *Scx* expression at the highest dose of bFGF (100 ng/mL) (Figure 3J), suggesting that the Prg4-GFP+ cells may be of tendon/ligament lineage.

Single cell RNA-seq identified similar cell populations in *in vitro* generated micromasses and mouse embryonic limb buds

To identify the cell types and developmental stages of Gdf5-RFP+ and Prg4-GFP+ cells, we performed single cell transcriptomic analysis of micromasses when reporter genes were expressed: TGF-β3, TGF-β3+PD (high Gdf5-RFP), and TGF-β3+bFGF (high Prg4-GFP) on day 7, and micromasses treated with TGF-β3 and TGF-β3+IWP2 (high Gdf5-RFP) on day 14. As *in vivo* controls, we sequenced cells isolated from mouse embryonic limb buds at the onset of *Gdf5* expression in the developing joints (E12.5 forelimb) and two days later when specialized joint cell types were arising (E14.5 hindlimb and the autopod of E14.5 hindlimb) (Table S1). Each dataset was first evaluated individually, and cell clusters were identified using differentially expressed genes (DEGs) and known marker genes (Figure S4). We identified diverse cell types (e.g., connective tissue, chondrogenic, myogenic, neural, limb bud mesenchyme, endothelial, among others) in the limb buds, as expected. *In vitro*, the most prominent cell types were chondrogenic, connective tissue-like, and cells that co-express mesoderm and chondrogenic genes (Alexandrovich et al., 2008; Almubarak et al., 2021) (Figures S4A and S4D). Small proportions of cells with neuronal or mitochondrial signatures were termed "off-target" (Figure S4; Table S1).

To comparatively analyze mESC-derived cells and mouse embryonic limb bud cells, we integrated the chondrogenic, connective tissue, and mesoderm/mesenchyme clusters from each sample (Figures 4A–4C). E11, E13, and E15 hindlimb bud cells, sequenced by Kelly et al. (2020) were also integrated to represent additional and intermediate time points of *in vivo* limb development. Six clusters were identified (Figures S5A and S5B; Table S2), reflecting chondrogenic cells (expressing *Col2a1*, *Acan*, *Col9a1*, *Col11a1*, and *Hapln1*), chondrogenic progenitor cells (expressing *Sox5*, *Sox6*, and *Sox9*) (Bhattaram et al., 2010), two distinct connective tissue populations (expressing *Col1a1*, *Col3a1*, *Dcn*, *Lum* or *Egfl6*, *Postn*, *Scx*, and *Tnmd*) (Markman et al., 2023; Ying et al., 1997; Raouf et al., 2002; Liu et al., 2015; Sakai and Kumagai, 2025), and limb bud mesenchyme/proliferative lineages (expressing *Prrx1*, *Lhx9*, *Mecom*, *Ebf1*, *Foxp1*, *Msx1*, and *Top2a*) (Leussink et al., 1995; Martin et al., 1995; Satoh et al., 2007; Tzchori et al., 2009) (Figures 4A–4D, and 4E). Chondrogenic cells were present in all *in vitro* treatments, suggesting that a chondrogenic fate was being induced as early as 1 week of culture

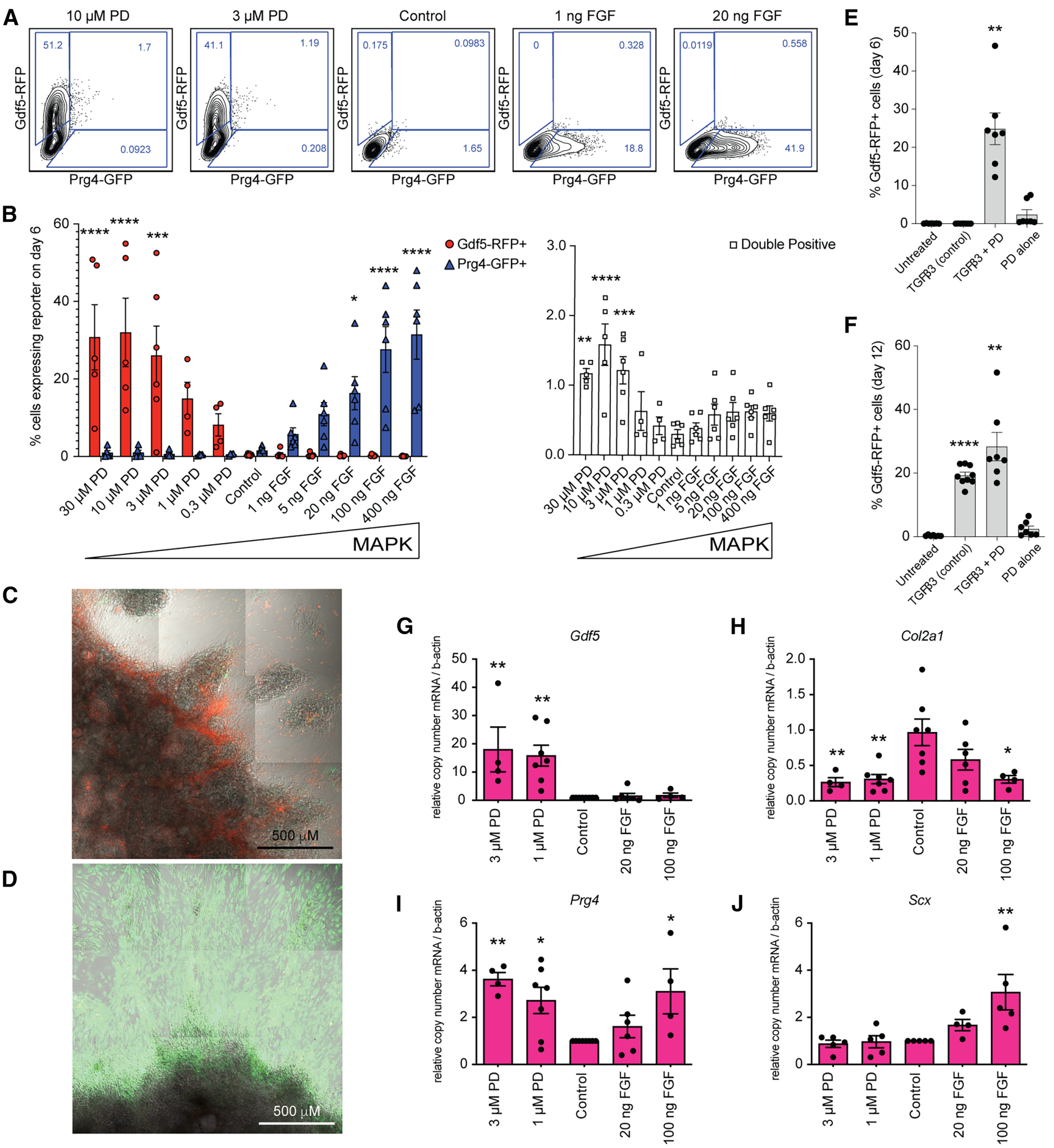

Figure 3. Modulation of MAPK signaling results in the expression of Gdf5-RFP and Prg4-GFP

(A) Representative flow cytometry contour plots show expression of Gdf5-RFP and Prg4-GFP on day 6 of micromass culture following indicated treatment. All treatments are in the presence of TGF-β3 (10 ng/mL). PD, PD0325901; control, TGF-β3 treatment alone.

(B) Percentage of Gdf5-RFP and/or Prg4-GFP expressing cells on day 6 of micromass culture in indicated conditions. Icons represent independent experiments. Values, mean ± SEM. ANOVA with Dunnett's multiple comparison correction, *p < 0.05, **p < 0.01, ***p < 0.001, and ****p < 0.0001 versus control.

(C) Confocal fluorescence and bright field microscopy image of TGF-β+PD (10 mM) treated micromass on day 6.

(D) Confocal fluorescence and bright field microscopy image of TGF-β+bFGF (20 ng/mL) treated micromass on day 6.

(E) Percentage of Gdf5-RFP expressing cells on day 6 of micromass culture following indicated treatment. Icons represent independent experiments. ANOVA with Dunnett's multiple comparison correction, **p < 0.01 versus control.

(F) Percentage of Gdf5-RFP expressing cells on day 12 of micromass culture following indicated treatment. Icons represent independent experiments. ANOVA with Dunnett's multiple comparison correction, **p < 0.01 and ****p < 0.0001 versus control.

(G–J) RT-qPCR quantified expression of *Gdf5* (G), *Prg4* (H), *Col2a1*(I), and *Scx* (J) on day 6 of micromass culture. Icons represent independent experiments. Values, mean ± SEM. ANOVA with Dunnett's multiple comparison correction, *p < 0.05 and **p < 0.01 versus control. See also Figure S3.

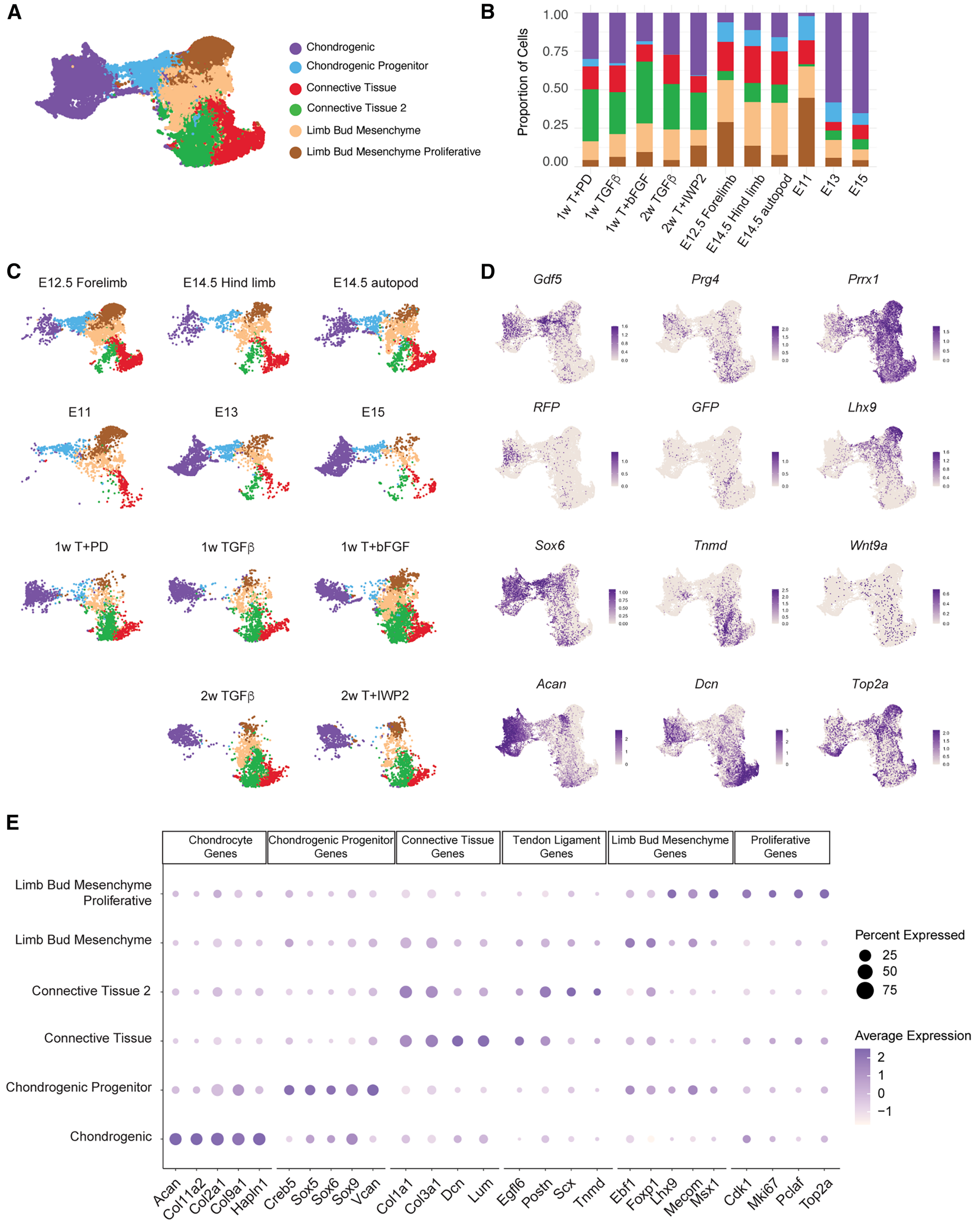

Figure 4. Overview of single cell RNA sequencing analysis

(A) Integrated UMAP of *in vitro* and *in vivo* datasets.

(B) Bar graph illustrates the proportion of each cluster per sample.

(C) Individual (split) UMAPs illustrate the general proportion of cells with each cell cluster. E11, E13, and E15 hindlimb samples were previously published (Kelly et al., 2020).

(D) Feature plots illustrate the level of indicated genes' expression in each cell in integrated RNA object.

(E) Dot plot shows the percent of cell expressing and the average normalized expression levels of indicated genes in each cluster of the integrated RNA object. See also Figure S4.

(Figure 4C). TGF-β3+IWP2 cultures proportionally had more chondrogenic cells compared to TGF-β-3 alone, consistent with the flow cytometry and gene expression data (Figure 4B). TGF-β3+PD (high Gdf5-RFP+) and TGF-β3+bFGF (high Prg4-GFP+) micromass cells after 1 week also showed contrasting cell composition, with PD treatment having increased chondrogenic cells, and bFGF treatment having proportionally more cells within the connective tissue 2, limb bud mesenchyme and limb bud mesenchyme proliferative clusters (Figure 4B).

Gdf5-expressing cells were largely clustered with chondrogenic progenitors and chondrocytes (Figure 4D). mESC-derived cells expressing *RFP* mRNA overlapped with cells expressing *Gdf5* mRNA, consistent with cell sorting (Figure 1F) and histological results (Figure 2G). *Gdf5* expression did not overlap significantly with the canonical Wnt ligand *Wnt9a*, which induced ectopic *Gdf5* expression in the developing limb (Guo et al., 2004) (Figure 4D). *Prg4*-expressing cells and *GFP*-expressing cells were found in all clusters, consistent with a variety of joint tissue lineages known to express *Prg4*. Notably, both *Prg4*+ and *GFP*+ cells are found in the connective tissue 2 cluster, which co-expressed *Tnmd* and *Scx* (Figures 4D and 4E). The specificity of *Prg4* expression to mESC-derived cells expressing GFP was confirmed following cell sorting and qPCR (Figure S2D).

We split the UMAPs by sample/treatment to specifically interrogate if changes in gene expression arose in one or more clusters following differential treatment (Figure 5A). While all *in vitro* cultures had cells with a limb bud mesenchyme-like signature, the TGF-β3+bFGF micromass had the highest number of cells expressing the limb bud, somite, and craniofacial mesenchyme gene *Msx1*. All cultures also contained cells within the two connective tissue clusters, however only TGF-β3+bFGF-treated cultures contained a large proportion of cells expressing the tendon/ligament-specific gene *Tnmd* (Figure 5A). The majority of *Tnmd* expressing cells were located within the connective tissue 2 cluster, which we subset from TGF-β3+PD (high Gdf5-RFP+) and TGF-β3+bFGF (high Prg4-GFP+) treatments to investigate changes in gene expression (Figure 5B). MAPK target genes such as *Dusp6*, *Spry1*, and *Etv1* (Janknecht, 1996; Hanafusa et al., 2002; Ahmad et al., 2018) were among the highest DEGs in the TGF-β3+bFGF treatment, along with *GFP*, consistent with flow cytometry data. Connective tissue 2 cells in the TGF-β3+PD treatment had significantly higher levels of *Gdf5* and *RFP*, as expected, and genes associated with ECM organization like *Lox* (Ahsan et al., 2005; Makris et al., 2013) and *Sparc* (Mundlos et al., 1992) and the glycoprotein *Cd248* (Hong et al., 2024). The chondrogenic cluster in TGF-β3 or TGF-β3+IWP2 micromasses were also specifically subset to quantify gene expression changes in the chondrocyte lineage (Figure 5C). *Col2a1*, *Col9a3*, *Acan*, and *Cnmd* were among the most significant genes elevated in the TGF-β3+IWP2 treatment (Figure 5C). While it remained clear that the cells within this cluster in the TGF-β3 micromass were also chondrocytes, genes more highly expressed encoded for ribosomal proteins.

Overall, these data identified similar cell populations within *in vitro* generated mESC-derived micromass cultures and mouse embryonic limb buds, highlighting the *in vivo* relevance of this *in vitro* experimental model and suggest we can use the mESC model to study these phenomena.

Effect of TGF-β, Wnt, and MAPK modulation in *ex vivo* embryonic limb bud cultures

In vitro cell culture can introduce artifacts that are not present *in vivo*, and likewise, there are signals present *in vivo* that are not precisely recapitulated *in vitro*. Given the transcriptional similarities of cells within mESC-derived micromasses and embryonic limb buds, we sought to determine whether signaling pathways we identified *in vitro* could induce *Gdf5* expression in primary cells *ex vivo* using a well-established limb bud micromass assay (Kulyk et al., 1989; Seo and Serra, 2007) (Figure 6A). We first assessed the baseline expression patterns of *Gdf5* in this *ex vivo* micromass culture using two different transgenic mice with LacZ reporter genes under the control of (1) a 200 kb BAC containing all known upstream and downstream regulatory elements of *Gdf5* (Chen et al., 2016; Capellini et al., 2017) or (2) an enhancer element, *R4*, that drives expression specifically in cells within the joint regions in developing mice, also referred to as PHC21 strain (Chen et al., 2016; Richard et al., 2020). Embryonic limb bud cells were isolated at E11.5, prior to the onset of *Gdf5* expression, and cultured in micromass (Figure 6A). Remarkably, LacZ expression patterns in the micromass cultures of each strain were reminiscent of their *in vivo* expression, where expression in the 200 kb BAC transgenic mouse at E14.5 was broader than that of the *Gdf5-R4-LacZ* transgenic mouse (Figures S6A and S6B). Micromasses from the 200 kb BAC transgenic mouse had LacZ+ cells primarily within chondrogenic nodes and less frequently in cells adjacent to and surrounding chondrogenic nodes (Figure S6B). The majority of *Gdf5-R4-LacZ*-derived positively stained cells, in contrast, were adjacent to and in between chondrogenic nodes (Figure S6B), similar to the specificity of this enhancer-reporter in developing joints *in vivo*. Thus, to understand the initiation of *Gdf5* expression in developing joint cells, we tested the effects of modulating TGF-β, Wnt, and MAPK signaling using the *Gdf5-R4-LacZ* mouse strain (Figure 6).

The initial requirement of TGF-β signaling Gdf5-RFP induction in mESC-derived cells suggested that TGF-β would increase the percentage of *Gdf5-R4-LacZ* expressing cells in

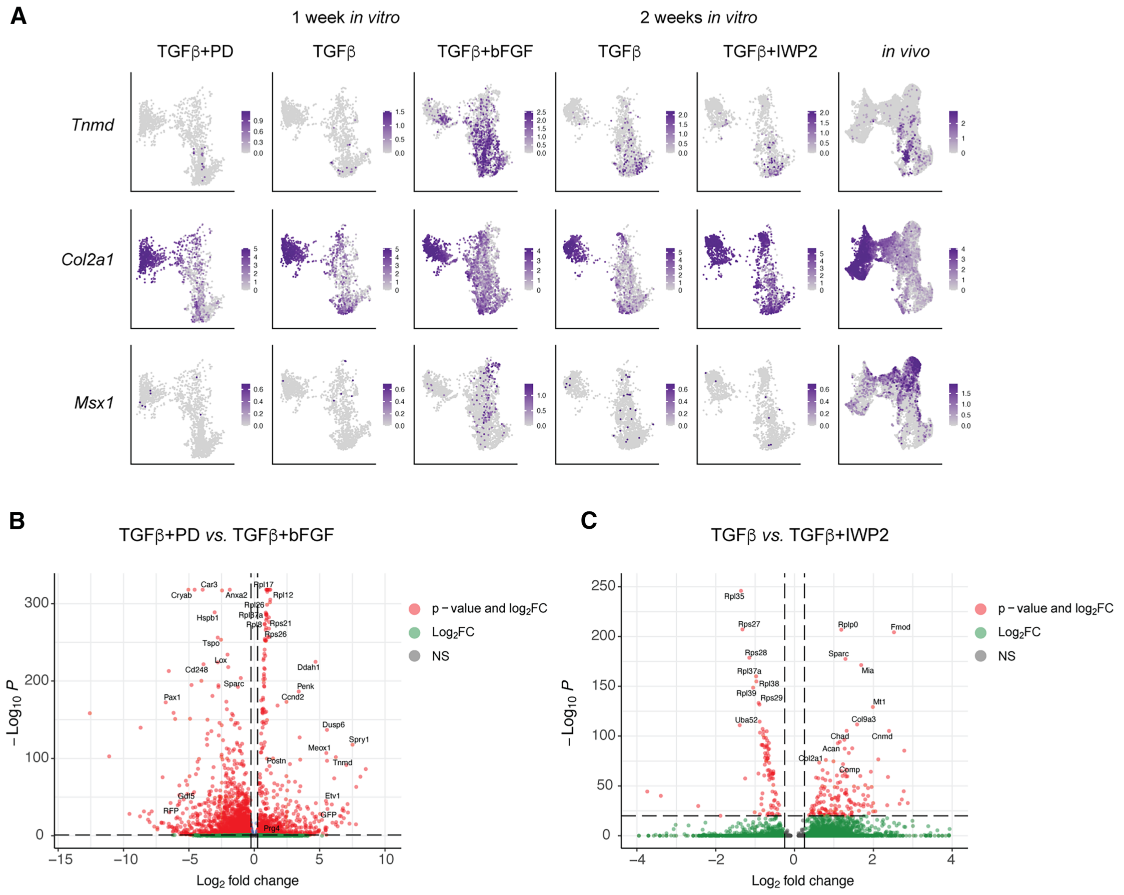

Figure 5. Transcriptomic changes that occur in mESC-derived micromass cultures as a result of treatment conditions
(A) Feature plots illustrate the level of indicated genes' expression in each cell in UMAPs split by sample.
(B) Volcano plot illustrates differentially expressed genes.
in the connective tissue 2 clusters from TGF-β+PD and TGF-β+bFGF samples. *Gdf5 and RFP* were enriched in cells treated with TGF-β+PD, while MAPK signaling target genes (e.g., *Dusp6*, *Etv1*, and *Spry1*) and genes associated with the tendon/ligament lineage (e.g., *Tnmd* and *Prg4*) were enriched in cells treated with TGF-β+bFGF. Cutoffs at log(2)FC >1 and *p*adj <0.005.
(C) Volcano plot illustrates differentially expressed genes in the chondrogenic clusters from TGF-β and TGF-β+IWP2 samples after two weeks of treatment. Chondrogenic genes such as *Col2a1*, *Comp*, *Cnmd*, and *Acan* are enriched in cells treated with TGF-β+IWP2. Cutoffs at log(2)FC > 1 and *p*adj <0.005. Genes encoding ribosomal proteins were not omitted from the datasets for DEG analyses. See also Figure S5.

the limb bud micromasses. However, a small but significant reduction in the number of LacZ+ cells was observed after 4 days compared to untreated controls (Figures 6B and 6C). This unexpected result could reflect the endogenous (i.e., sufficient) levels of TGF-β signaling in the chondrogenic mesenchyme in E11.5 limb buds or the differences in regulatory control of the *R4* enhancer versus the endogenous *Gdf5* locus. The expression of *Gdf5* mRNA in TGF-β-treated micromasses was not significantly different from control (Figure 6D), supporting the latter. To address the former, we quantified the expression of several genes known to be regulated by TGF-β signaling in isolated limb bud cells compared to mESC-derived chondrogenic mesoderm cells prior to and after TGF-β treatment in micromass (Figures S6C and S6D). The expression of *Snai1* (Basu et al., 2015), *Ccn2* (Igarashi et al., 1993; Untergasser et al., 2005), *Pai1* (Geng et al., 1999; Hirashima et al., 2003), and *Smad7* (Afrakhte et al., 1998) were all significantly higher in E11.5 limb bud cells compared to chondrogenic mesoderm cells on day 5 of differentiation (Figure S6C). Following 6 days in micromass with TGF-β, mESC-derived cells upregulated all 4 genes, and only *Pai1* was significantly different from the E11.5 limb bud cells, being higher in the mESC-derived cells (Figure S6D). These results suggest that endogenous TGF-β signaling was sufficient to permit expression of Gdf5-R4-LacZ *ex vivo*; therefore, we tested the effects of modulating Wnt and MAPK signaling in the absence of exogenous TGF-β.

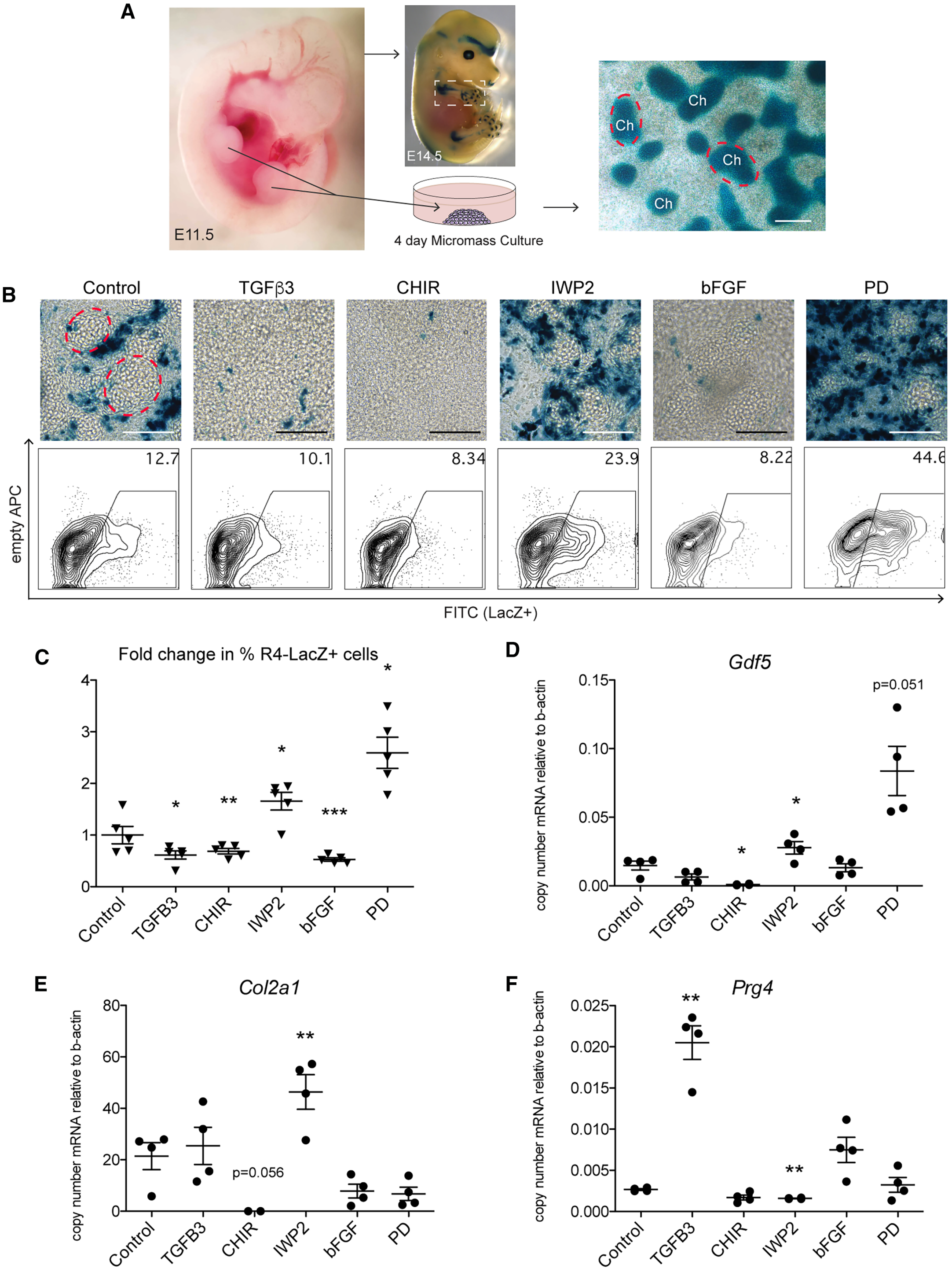

Figure 6. Gdf5-R4-LacZ reporter expression and chondrogenesis in embryonic limb bud micromass cultures following TGF-β, Wnt, and MAPK modulation

(A) Limb buds from Gdf5-R4-LacZ transgenic mice were microdissected on embryonic day 11.5 (E11.5) and isolated cells were cultured in micromass for 4 days in the presence or absence of factors: TGF-β3 10 ng/mL, CHIR 1 μM, IWP2 2 μM, bFGF 20 ng/mL, and PD 3 μM. Chondrogenic nodes (subset indicated with dashed red line and/or “Ch”) in limb bud micromass cultures stain positively with Alcian blue indicating presence of proteoglycans/glycosaminoglycans in cartilage matrix. Scale bars, 200 μm.

(legend continued on next page)

Limb bud cells isolated from 5 independent litters were treated in micromass with CHIR (1 μM), IWP2 (2 μM), bFGF (20 ng/mL), or PD (3 μM), and the expression of *Gdf5-R4-LacZ* was quantified by flow cytometry and microscopy on day 4 (Figures 6B and 6C). Similar to the results observed in mESC-derived micromasses, activation of Wnt with CHIR or MAPK with bFGF significantly reduced the percentage of *Gdf5-R4-LacZ*+ cells while inhibition of Wnt with IWP2 or inhibition of MAPK signaling with PD significantly increased the percentage of *Gdf5-R4-LacZ*+ cells (Figure 6C). *Gdf5* expression was increased in replicate cultures from each litter with IWP2 and PD treatment (Figure 6D), with PD being significantly higher than control. CHIR significantly reduced *Gdf5* expression, and no significant changes were observed with TGF-β or bFGF treatment. Similar to the chondrogenic-promoting effects of Wnt inhibition in mESCs, IWP2 treatment led to a significant increase in *Col2a1* in the limb bud cells, which was reduced in the CHIR treatment (Figure 6E). We confirmed, in non-transgenic limb bud cells, that recombinant proteins that activate (Wnt3a) or inhibit Wnt (DKK1) behaved similarly to the small molecules used in this study (Figure S6E). bFGF and PD treatments both resulted in decreases in *Col2a1* compared to untreated controls, but not significantly (Figure 6F). Finally, as expected, TGF-β3 and bFGF treatments both increased *Prg4* expression (Figure 6G), with the TGF-β3-mediated increase being significant. These data support the notion that signals we identified to induce *Gdf5* and *Prg4* expression in mESCs *in vitro* were able to induce similar expression and cell fate changes in mouse embryonic limb bud cells *ex vivo*.

DISCUSSION

In this study, we generated a dual fluorescent reporter mESC line to study the role of key signaling pathways in joint development. We utilized directed differentiation methodology and demonstrated that TGF-β signaling is necessary for induction of *Gdf5* expression *in vitro*, and modulation of Wnt and MAPK signaling pathways significantly impacted the onset of both *Gdf5* and *Prg4* expression. Single cell transcriptomic analysis revealed that joint lineage cells induced by TGF-β, Wnt, and MAPK modulation had similar transcriptomic profiles to chondrogenic and connective tissue lineages in mouse embryonic limb buds. Finally, we corroborated our *in vitro* findings regarding modulation of these pathways in *ex vivo* limb bud cultures, highlighting the utility of this dual reporter mESC line in studying specification of joint progenitor cells and joint developmental processes.

Our finding that canonical Wnt signaling prohibits chondrogenesis in both mESC and limb bud micromasses is consistent with previous reports (Koyama et al., 1996; Serra et al., 1997; Wu et al., 2021). Building on this, we found that inhibition of Wnt promoted *Gdf5* expression and chondrogenesis, which upon face value is inconsistent with previous reports of Wnt9a/14 ligand expression in the interzone, and that ectopic Wnt9a/14 expression can induce *Gdf5* expression (Guo et al., 2004; Hartmann and Tabin, 2001; Bhattaram et al., 2014). We suggest these data support a dynamic temporal role for Wnt signaling in joint development. *In vivo*, Wnt activation may be required to suppress chondrogenesis, as suggested by high endogenous expression of TGF-β target genes and the fact that exogenous TGF-β was not necessary for *Gdf5* expression *ex vivo* as it was *in vitro*. At later times, Wnt signaling must be dampened to support chondrogenesis of those same progenitors. *In vitro*, however, the Wnt-activating function of chondrogenic repression was not required, as suggested by significantly lower basal levels of TGF-β target genes and the requirement of exogenous TGF-β to induce *Gdf5* expression and chondrogenesis.

MAPK inhibition, via MEK inhibition, strongly promoted the expression of *Gdf5* in both mESC-derived and *ex vivo* limb bud cultures, a finding that provides additional insights around joint cavity formation. The joint line that will ultimately result in the creation of the joint cavity is known to be driven through the activation of the MEK-ERK pathway (Bastow et al., 2005), and this niche inhibits *Gdf5* expression to prevent joint fusion (Lamb et al., 2003). The role of joint line cells in promoting cavitation is thus distinguishable from the *Gdf5*-expressing cells that will emerge and undergo differentiation. The former exhibit activated MEK/ERK, while the latter present with decreased MEK/ERK signaling. This phenomenon is consistent with the observed acceleration of *Gdf5* expression and

(B) Gdf5-R4-LacZ-expressing cells were visualized by X-gal staining of micromass cultures after 4 days. Dashed red lines in the control image indicate representative chondrogenic nodes. Scale bars, 75 μm. Representative contour plots indicate the percentage of cells expressing the LacZ reporter quantified by flow cytometry using the FDG reagent.
(C) Fold change in the percentage of Gdf5-R4-LacZ-expressing cells within each litter (n) is depicted. Each treatment value was normalized to untreated control culture within the same litter, $n = 5$ litters (independent experiments). Values, mean ± SEM. ANOVA with Dunnett's multiple comparison correction. *$p < 0.05$, **$p < 0.01$, and ***$p < 0.001$ vs. control.
(D–F) RT-qPCR of *Gdf5*, *Col2a1*, and *Prg4* following 4 days of micromass culture, $n = 4$ litters (independent experiments), Values, mean ± SEM. ANOVA with Dunnett's multiple comparison correction. *$p < 0.05$ and **$p < 0.01$. See also Figure S6.

chondrogenesis upon MEK inhibition *in vitro* and *ex vivo* in this study.

Lineage tracing in mice have reproducibly shown that Gdf5-lineage cells contribute to the formation of permanent articular cartilage, and not to the chondrogenic mesenchyme/growth plate cartilage involved in endochondral ossification (Koyama et al., 2008; Shwartz et al., 2016). It is thus justly hypothesized that Gdf5-lineage cells would be an optimal progenitor cell source to not only study intra-articular tissue development, but to generate permanent articular cartilage cells and tissues for replacement or repair. hESC-derived populations enriched for *GDF5* expression preferentially generated chondrocytes lacking a growth plate-like phenotype compared to those enriched for *SOX9* expression (Pothiawala et al., 2022). We performed similar experiments in which we demonstrated that articular chondrocytes derived from hESCs, which expressed *GDF5* at earlier time points, remained cartilaginous *in vivo*, and did not initiate ossification following subcutaneous injection in mice (Craft et al., 2015). Following implantation of these same articular cartilage tissues in focal cartilage defects in the rat, however, regions exposed to bone marrow spaces did undergo remodeling and hypertrophy as one might expect from a more naive cartilage anlage (Gardner et al., 2019). In earlier studies using chondrocytes derived from mESCs, we found ossification was only prevented when grafts were physically protected from invading host vasculature (Craft et al., 2013). In contrast, GDF5 also participates in promoting vascularization in a rabbit long-bone defect model (Kleinschmidt et al., 2014), and both recombinant GDF5 (Coleman and Tuan, 2003) and forced expression of *GDF5* can promote chondrocyte hypertrophy (Tsumaki et al., 1999). Thus, we do not expect that chondrocytes enriched in *Gdf5/GDF5* expression will remain as permanent cartilage in a scenario where vascularization is permitted or if *Gdf5/GDF5* expression persists. The optimal progenitor cell is important, but the recipient environment must be optimal as well.

Through our manipulation of the MAPK pathway, we observed that treatment with basic bFGF increased expression of Prg4-GFP and *Prg4* mRNA in a dose dependent manner, concomitant with evidence of a tendon/ligament lineage. Expression of *Prg4* mRNA was also increased in PD-treated cultures to the same extent, while these cultures lacked a significant proportion of Prg4-GFP+ cells. *Prg4* expression in PD-treated cultures could reflect the higher percentage of double-positive cells, but it is possible that reporter expression is not perfectly aligned with endogenous gene expression from the untargeted allele, raising concerns about the conclusions we have made. In a recent study using a complementary *Col2a1-IRES-nGFP;Prg4-tdTomato* mESC reporter line, we found *Prg4*, in a scenario where *Col2a1* expression is absent, to be a useful surrogate marker for tendon-like cells (Niu et al., 2024). In support of our findings here, activation of MAPK in mESC-derived progenitors with either cyclic (c)AMP agonists or bFGF in this study also resulted in the generation of *Prg4*-expressing cells that expressed tendon/ligament gene repertoires (e.g., *Scx* and *Tnmd*). Thus, while we showed that MAPK plays a role in promoting the tendon-ligament lineage, further investigation is needed to delineate the discrepancy between the GFP reporter and *Prg4* mRNA expression in this mESC line.

Synovial joints are present in both the appendicular and axial skeleton and prior work has shown that regulatory elements within the upstream BAC and downstream 200 kB BAC drive Gdf5-lacZ expression in both anatomical locations in mice (Chen et al., 2016). *In vitro*, directed differentiation methodologies to induce paraxial mesoderm, which gives rise to cartilage in the vertebrae and ribs, versus lateral plate mesoderm, responsible for cartilage formation in the limbs, have become increasingly refined over time (Chal et al., 2018; Pothiawala et al., 2022; Kattman et al., 2011; Loh et al., 2016). Paraxial mesoderm, often induced with Wnt activation and BMP inhibition, can be identified in *in vitro* cultures by the expression of Pdgfra/PDGFRA and lack of Flk-1/KDR, while lateral plate mesoderm induction protocols include active BMP signaling, resulting in the expression of both of these markers. However, the important distinction between articular cartilage, which is derived from a joint cell progenitor, and growth plate cartilage, which is not (Koyama et al., 2008) has been more extensively described using paraxial mesoderm induction protocols (Craft et al., 2015; Pothiawala et al., 2022). The protocol used herein also induced a Pdgfra^{+}Flk-1^{-} mesoderm population with BMP inhibition and endogenous Wnt signaling, suggesting a paraxial mesoderm intermediate, although this was not shown definitively. Thus, we cannot claim that cells expressing Gdf5-RFP or Prg4-GFP are of the same anatomical origin as the *ex vivo* limb bud cells. We did however corroborate findings from the *in vitro* micromass culture to *ex vivo* mouse embryonic limb bud cultures under a number of different treatment conditions. These results suggest that there may be shared mechanisms of joint cell specification in developing axial and appendicular synovial joints, and this ESC model may recapitulate fundamental concepts regarding the molecular regulation of this process.

The epigenetic regulation of *Gdf5* expression is anatomically complex, and one caveat to this study is that the expression of the fluorescent reporters in the mESC line was not confirmed to track with gene expression in the mouse embryo. The *Gdf5 R4* enhancer element resides within a much larger regulatory locus, encapsulated by at least 200 kb of genomic sequence containing many

additional tissue-specific regulatory elements, including *GROW1*, which activates Gdf5 in chondrogenic long bone/growth plate regions (Capellini et al., 2017; Muthuirulan et al., 2021; Chen et al., 2016; Coveney et al., 2025). It was remarkable to find that the pattern of *LacZ* expression driven by the larger 200 KB (BAC) reflected a broader domain, as expression resided both adjacent to (as driven by the *R4* sequence) and within chondrogenic nodes. *In vivo*, the *R4* regulatory element drives strong *Gdf5* and *LacZ* expression in joint interzones and then in the developing joint tissues through early post-natal life, which correlates with its expression in cells adjacent to and along the periphery of the chondrogenic nodes of limb bud micromasses. Interestingly, loss of this enhancer in mice and human cells alters joint morphology and contributes to osteoarthritis risk (Richard et al., 2020). These regulatory elements and their specific expression domains were also identified during human chondrogenesis and joint formation (Richard et al., 2020; Muthuirulan et al., 2021), indicating that such signal-regulatory element interactions are conserved across mammals, and likely across amniotes.

Since the discovery of *Gdf5* as the earliest marker of joint initiation (Storm and Kingsley, 1999; Koyama et al., 2008), we have made limited progress in deciphering the cues underlying the specification of these progenitor cells that give rise to joint tissues required for mobility. As injuries to joint tissues are prevalent in all demographics, it is important that we investigate these developmental processes to support the evolution of novel therapeutics. This study introduced new experimental platforms that successfully elucidated mechanisms for promoting joint cell identities, thereby illuminating a poorly understood area of cell fate determination and furthers our ability to generate joint lineage cells from pluripotent stem cells.

METHODS

Generation of dual mESC reporter line

The coding sequences for tdRFP and eGFP were introduced into the Gdf5 and Prg4 loci, respectively, of E14 mESCs using CRISPR-Cas9 (Cong et al., 2013) to generate the BC29 dual reporter line (Figures S1A and S1B). Clones were confirmed by Sanger sequencing. We note that the untargeted allele of Prg4 is missing one cysteine codon in the signal peptide.

ESC maintenance and differentiation

mESCs were maintained in a modified serum-free (SF), feeder-free culture system (i.e., 2i media) as described previously (Sim et al., 2017; Craft et al., 2013; Gadue et al., 2006; Ying et al., 2003). For differentiation, ESCs were cultured in suspension in SF differentiation medium (SFD) (Gouon-Evans et al., 2006) without additional factors for 48 h. Embryoid bodies were treated with activin A, CHIR99021, and noggin or LDN-193189 for 28 h to induce primitive streak mesoderm, then resuspended in SFD containing bFGF and Y-27632 for 48 h. High density micromasses were generated from mesoderm cells on day 5 (Craft et al., 2015) and maintained in chondrogenic media consisting of high glucose DMEM with 1× Insulin Transferrin Selenium (ITS) supplement, ascorbic acid (50 μg/mL), proline (40 μg/mL), and dexamethasone (0.1 μM) with indicated factors. Recombinant growth factors and small molecules were purchased from R&D Systems and Sigma-Aldrich. Details including concentrations of all factors are in the supplemental information.

Mouse embryonic limb bud cultures

Animal studies were performed in compliance of ethical regulations and were approved by Animal Resources at Children's Hospital or Harvard University. Wild-type FVB (friend virus B) strain mice, transgenic 200 kb BAC Gdf5 LacZ mice, and *PHC21* (*Gdf5-R4-LacZ*) mice (both FVB strain) were used for these studies. From each pregnant dam, forelimb and hindlimb buds were micro-dissected from all mouse embryos, pooled prior to dissociation with 0.2% type I collagenase for two hours at 37°C and cells were plated in micromass.

Flow cytometry and cell sorting

The following antibodies were used for flow cytometry: anti-mouse Flk-1-biotin, anti-mouse Pdgfrα (CD140a)-allophycocyanin (APC; clone APA5), streptavidin-phycoerythrin (PE) or streptavidin-PE-Cy7 (BD Pharmingen). Micromasses were dissociated with collagenase prior to analysis. Limb bud cells expressing beta-galactosidase (*Gdf5-R4-LacZ* transgene) were detected using the fluorescein di-V-galactoside (FDG) reagent within the FluoReporter LacZ flow cytometry kit (F1930, Thermo Fisher Scientific) following manufacturer's instructions. Cells were analyzed or sorted using a BD FACS Fortessa and BD Melody (Becton Dickinson), analysis was performed using FlowJo (Tree Star), and mean, standard errors and all statistical tests were calculated in Prism (GraphPad).

Quantitative reverse-transcription PCR (RT-qPCR)

Total RNA was extracted using the MagMAX mirVana Total RNA kit (Applied Biosystems), and reverse transcribed with Superscript IV VILO reverse transcriptase with ezDNase enzyme (Invitrogen). RT-qPCR was performed on a ViiA 7 Real-Time PCR System using PowerUp SYBR Green PCR kit (Applied Biosystems). The copy number of each gene shown is normalized to that of *β-actin*. Biological replicates/independent experiments are indicated

in figures or legends. Primer sequences are in the supplemental material. Mean, standard errors, and all statistical tests were calculated in Prism (GraphPad).

Histology, staining, and imaging

Micromasses from limb bud cells were fixed in 4% paraformaldehyde (PFA) prior to standard X-gal and Alcian blue staining. 5–10 μm sections of micromasses derived from mESCs were cryosectioned, fixed in 4% PFA, and mounted with DAPI-containing media or stained with toluidine blue. Imaging was performed on an EVOS FL Auto 2 or a Zeiss LCM800 confocal microscope.

scRNA-seq methods and analysis

scRNA-seq libraries were prepared using Single Cell 3′ Library & Gel Bead Kit v2 (10× Genomics) and sequenced on an Illumina NextSeq 500 to ∼30,000 reads per cell. Reads were processed by Cell Ranger (version 3.0.0), and aligned to the GRCm38 (version 100) genome amended with RFP and eGFP transgenes. Cells with <500 UMIs, >70,000 UMIs, or >20% mitochondrial DNA were removed. The cell matrix was normalized using SCTransform (Hafemeister and Satija, 2019), and uniform manifold approximation and projection (UMAP) was used for dimensional reduction and visualization (McInnes et al., 2018). Clustering and DEGs were defined using Leiden algorithm (Traag et al., 2019) and the model-based analysis of single-cell transcriptomics (MAST) framework (Finak et al., 2015). Indicated cell types were integrated using reciprocal PCA (Butler et al., 2018), using the E14.5 limb samples as reference. Details including all packages and versions used can be found in the supplemental information.

RESOURCE AVAILABILITY

Lead contact

Requests for further information and resources should be directed to and will be fulfilled by the lead contact, April Craft (april.craft@childrens.harvard.edu).

Materials availability

Plasmids and the mESC line generated in this study are available for research purposes with a completed materials transfer agreement.

Data and code availability

Raw single-cell transcriptomic data used in this publication are deposited at GEO: GSE274060. All code and analyzed R objects are available on Github at github.com/suyraj/Mouse-Cartilage-scRNAseq/tree/main. Requests for additional information should be directed to the corresponding author.

ACKNOWLEDGMENTS

The authors would like to acknowledge Dr. Patrick Tschopp (University of Basel) for thoughtful discussions regarding the research project, and Dr. Clarissa Coveney and members of the Craft lab for critical reading of the manuscript. This project was supported with NIH/NIAMS R01-AR073821 [A.M.C.] and R01-AR070139 [T. D.C.] and Arthritis National Research Foundation (A.M.C.) funding. We thank the BPF Genomics Core Facility at Harvard Medical School (RRID: SCR_007175) and the Center for Musculoskeletal Research (P30 AR075042) for expertise that supported this work.

AUTHOR CONTRIBUTIONS

Conceptualization, A.M.C.; formal analysis, S.R., T.C., A.M.C., and M.A.-R.; funding acquisition, A.M.C. and T.D.C.; investigation, S.R., T.C., S.M., M.A.-R., J.C., and S.K.J., methodology, S.R., T.C., and A.M.C.; resources, S.R., M.Y., and T.D.C.; visualization, S.R., T.C., and A.M.C.; writing – original draft, S.R., S.K.J., and A.M.C.; writing – review & editing, S.R., S.K.J., T.D.C., and A.M.C.

DECLARATION OF INTERESTS

The authors declare no competing interests.

SUPPLEMENTAL INFORMATION

Supplemental information can be found online at https://doi.org/10.1016/j.stemcr.2025.102685.

Received: November 20, 2024
Revised: September 28, 2025
Accepted: September 29, 2025
Published: October 23, 2025

REFERENCES

Afrakhte, M., Morén, A., Jossan, S., Itoh, S., Sampath, K., Westermark, B., Heldin, C.H., Heldin, N.E., and ten Dijke, P. (1998). Induction of inhibitory Smad6 and Smad7 mRNA by TGF-beta family members. Biochem. Biophys. Res. Commun. *249*, 505–511.

Ahmad, M.K., Abdollah, N.A., Shafie, N.H., Yusof, N.M., and Razak, S.R.A. (2018). Dual-specificity phosphatase 6 (DUSP6): a review of its molecular characteristics and clinical relevance in cancer. Cancer Biol. Med. *15*, 14–28.

Ahsan, T., Harwood, F., McGowan, K.B., Amiel, D., and Sah, R.L. (2005). Kinetics of collagen crosslinking in adult bovine articular cartilage. Osteoarthr. Cartil. *13*, 709–715.

Alexandrovich, A., Qureishi, A., Coudert, A.E., Zhang, L., Grigoriadis, A.E., Shah, A.M., Brewer, A.C., and Pizzey, J.A. (2008). A role for GATA-6 in vertebrate chondrogenesis. Dev. Biol. *314*, 457–470.

Almubarak, A., Lavy, R., Srnic, N., Hu, Y., Maripuri, D.P., Kume, T., and Berry, F.B. (2021). Loss of Foxc1 and Foxc2 function in chondroprogenitor cells disrupts endochondral ossification. J. Biol. Chem. *297*, 101020.

Bastow, E.R., Lamb, K.J., Lewthwaite, J.C., Osborne, A.C., Kavanagh, E., Wheeler-Jones, C.P.D., and Pitsillides, A.A. (2005). Selective Activation of the MEK-ERK Pathway Is Regulated by Mechanical Stimuli in Forming Joints and Promotes Pericellular Matrix Formation. J. Biol. Chem. *280*, 11749–11758.

Basu, M., Bhattacharya, R., Ray, U., Mukhopadhyay, S., Chatterjee, U., and Roy, S.S. (2015). Invasion of ovarian cancer cells is

induced byPITX2-mediated activation of TGF-β and Activin-A. Mol. Cancer *14*, 162.

Bhattaram, P., Penzo-Méndez, A., Kato, K., Bandyopadhyay, K., Gadi, A., Taketo, M.M., and Lefebvre, V. (2014). SOXC proteins amplify canonical WNT signaling to secure nonchondrocytic fates in skeletogenesis. J. Cell Biol. *207*, 657–671.

Bhattaram, P., Penzo-Méndez, A., Sock, E., Colmenares, C., Kaneko, K.J., Vassilev, A., DePamphilis, M.L., Wegner, M., and Lefebvre, V. (2010). Organogenesis relies on SoxC transcription factors for the survival of neural and mesenchymal progenitors. Nat. Commun. *1*, 9.

Brent, A.E., and Tabin, C.J. (2004). FGF acts directly on the somitic tendon progenitors through the Ets transcription factors Pea3 and Erm to regulate scleraxis expression. Dev. Camb. Engl. *131*, 3885–3896.

Brunet, L.J., McMahon, J.A., McMahon, A.P., and Harland, R.M. (1998). Noggin, Cartilage Morphogenesis, and Joint Formation in the Mammalian Skeleton. Science *280*, 1455–1457.

Butler, A., Hoffman, P., Smibert, P., Papalexi, E., and Satija, R. (2018). Integrating single-cell transcriptomic data across different conditions, technologies, and species. Nat. Biotechnol. *36*, 411–420.

Capellini, T.D., Chen, H., Cao, J., Doxey, A.C., Kiapour, A.M., Schoor, M., and Kingsley, D.M. (2017). Ancient selection for derived alleles at a GDF5 enhancer influencing human growth and osteoarthritis risk. Nat. Genet. *49*, 1202–1210.

Chal, J., Al Tanoury, Z., Oginuma, M., Moncuquet, P., Gobert, B., Miyanari, A., Tassy, O., Guevara, G., Hubaud, A., Bera, A., et al. (2018). Recapitulating early development of mouse musculoskeletal precursors of the paraxial mesoderm in vitro. Development *145*, dev157339.

Chen, H., Capellini, T.D., Schoor, M., Mortlock, D.P., Reddi, A.H., and Kingsley, D.M. (2016). Heads, Shoulders, Elbows, Knees, and Toes: Modular Gdf5 Enhancers Control Different Joints in the Vertebrate Skeleton. PLoS Genet. *12*, e1006454.

Coleman, C.M., and Tuan, R.S. (2003). Growth/differentiation factor 5 enhances chondrocyte maturation. Dev. Dyn. *228*, 208–216.

Cong, L., Ran, F.A., Cox, D., Lin, S., Barretto, R., Habib, N., Hsu, P.D., Wu, X., Jiang, W., Marraffini, L.A., and Zhang, F. (2013). Multiplex genome engineering using CRISPR/Cas systems. Science *339*, 819–823.

Coveney, C.R., Maridas, D., Chen, H., Muthuirulan, P., Liu, Z., Jagoda, E., Yarlagadda, S., Movahhedi, M., Proffen, B., Dashtdar, B., et al. (2025). Complex regulatory interactions at GDF5 shape joint morphology and osteoarthritis disease risk. Arthritis Rheumatol. https://doi.org/10.1002/art.43231.

Craft, A.M., Ahmed, N., Rockel, J.S., Baht, G.S., Alman, B.A., Kandel, R.A., Grigoriadis, A.E., and Keller, G.M. (2013). Specification of chondrocytes and cartilage tissues from embryonic stem cells. Development *140*, 2597–2610.

Craft, A.M., Rockel, J.S., Nartiss, Y., Kandel, R.A., Alman, B.A., and Keller, G.M. (2015). Generation of articular chondrocytes from human pluripotent stem cells. Nat. Biotechnol. *33*, 638–645.

Finak, G., McDavid, A., Yajima, M., Deng, J., Gersuk, V., Shalek, A.K., Slichter, C.K., Miller, H.W., McElrath, M.J., Prlic, M., et al. (2015). MAST: a flexible statistical framework for assessing transcriptional changes and characterizing heterogeneity in single-cell RNA sequencing data. Genome Biol. *16*, 278.

Gadue, P., Huber, T.L., Paddison, P.J., and Keller, G.M. (2006). Wnt and TGF-beta signaling are required for the induction of an in vitro model of primitive streak formation using embryonic stem cells. Proc. Natl. Acad. Sci. USA *103*, 16806–16811.

Gardner, O.F., Juneja, S.C., Whetstone, H., Nartiss, Y., Sieker, J.T., Veillette, C., Keller, G.M., and Craft, A.M. (2019). Effective repair of articular cartilage using human pluripotent stem cell-derived tissue. Eur. Cell. Mater. *38*, 215–227.

Geng, M.M., Ellenrieder, V., Wallrapp, C., Müller-Pillasch, F., Sommer, G., Adler, G., and Gress, T.M. (1999). Use of representational difference analysis to study the effect of TGFB on the expression profile of a pancreatic cancer cell line. Genes Chromosomes Cancer *26*, 70–79.

Gouon-Evans, V., Boussemart, L., Gadue, P., Nierhoff, D., Koehler, C.I., Kubo, A., Shafritz, D.A., and Keller, G. (2006). BMP-4 is required for hepatic specification of mouse embryonic stem cell-derived definitive endoderm. Nat. Biotechnol. *24*, 1402–1411.

Guo, X., Day, T.F., Jiang, X., Garrett-Beal, L., Topol, L., and Yang, Y. (2004). Wnt/β-catenin signaling is sufficient and necessary for synovial joint formation. Genes Dev. *18*, 2404–2417.

Hafemeister, C., and Satija, R. (2019). Normalization and variance stabilization of single-cell RNA-seq data using regularized negative binomial regression. Genome Biol. *20*, 296.

Hanafusa, H., Torii, S., Yasunaga, T., and Nishida, E. (2002). Sprouty1 and Sprouty2 provide a control mechanism for the Ras/MAPK signalling pathway. Nat. Cell Biol. *4*, 850–858.

Hartmann, C., and Tabin, C.J. (2001). Wnt-14 Plays a Pivotal Role in Inducing Synovial Joint Formation in the Developing Appendicular Skeleton. Cell *104*, 341–351.

Havis, E., Bonnin, M.-A., Esteves de Lima, J., Charvet, B., Milet, C., and Duprez, D. (2016). TGFβ and FGF promote tendon progenitor fate and act downstream of muscle contraction to regulate tendon differentiation during chick limb development. Development *143*, 3839–3851.

Hirashima, Y., Kobayashi, H., Suzuki, M., Tanaka, Y., Kanayama, N., and Terao, T. (2003). Transforming growth factor-beta1 produced by ovarian cancer cell line HRA stimulates attachment and invasion through an up-regulation of plasminogen activator inhibitor type-1 in human peritoneal mesothelial cells. J. Biol. Chem. *278*, 26793–26802.

Hong, Y.-K., Lin, Y.-C., Cheng, T.-L., Lai, C.-H., Chang, Y.-H., Huang, Y.-L., Hung, C.-Y., Wu, C.-H., Hung, K.-S., Ku, Y.-C., et al. (2024). TEM1/endosialin/CD248 promotes pathologic scarring and TGF-β activity through its receptor stability in dermal fibroblasts. J. Biomed. Sci. *31*, 12.

Igarashi, A., Okochi, H., Bradham, D.M., and Grotendorst, G.R. (1993). Regulation of connective tissue growth factor gene expression in human skin fibroblasts and during wound repair. Mol. Biol. Cell *4*, 637–645.

Janknecht, R. (1996). Analysis of the ERK-stimulated ETS transcription factor ER81. Mol. Cell Biol. *16*, 1550–1556.

Jay, G.D., Britt, D.E., and Cha, C.J. (2000). Lubricin is a product of megakaryocyte stimulating factor gene expression by human synovial fibroblasts. J. Rheumatol. *27*, 594–600.

Kattman, S.J., Witty, A.D., Gagliardi, M., Dubois, N.C., Niapour, M., Hotta, A., Ellis, J., and Keller, G. (2011). Stage-specific optimization of activin/nodal and BMP signaling promotes cardiac differentiation of mouse and human pluripotent stem cell lines. Cell Stem Cell *8*, 228–240.

Kelly, N.H., Huynh, N.P.T., and Guilak, F. (2020). Single cell RNA-sequencing reveals cellular heterogeneity and trajectories of lineage specification during murine embryonic limb development. Matrix Biol. *89*, 1–10.

Khalafi, A., Schmid, T.M., Neu, C., and Reddi, A.H. (2007). Increased accumulation of superficial zone protein (SZP) in articular cartilage in response to bone morphogenetic protein-7 and growth factors. J. Orthop. Res. *25*, 293–303.

Kleinschmidt, K., Wagner-Ecker, M., Bartek, B., Holschbach, J., and Richter, W. (2014). Superior angiogenic potential of GDF-5 and GDF-5(V453/V456) compared with BMP-2 in a rabbit long-bone defect model. J. Bone Joint Surg. Am. *96*, 1699–1707.

Koyama, E., Leatherman, J.L., Noji, S., and Pacifici, M. (1996). Early chick limb cartilaginous elements possess polarizing activity and express hedgehog-related morphogenetic factors. Dev. Dyn. *207*, 344–354.

Koyama, E., Shibukawa, Y., Nagayama, M., Sugito, H., Young, B., Yuasa, T., Okabe, T., Ochiai, T., Kamiya, N., Rountree, R.B., et al. (2008). A distinct cohort of progenitor cells participates in synovial joint and articular cartilage formation during mouse limb skeletogenesis. Dev. Biol. *316*, 62–73.

Kulyk, W.M., Rodgers, B.J., Greer, K., and Kosher, R.A. (1989). Promotion of embryonic chick limb cartilage differentiation by transforming growth factor-beta. Dev. Biol. *135*, 424–430.

Lamb, K.J., Lewthwaite, J.C., Bastow, E.R., and Pitsillides, A.A. (2003). Defining boundaries during joint cavity formation: going out on a limb. Int. J. Exp. Pathol. *84*, 55–67.

Leussink, B., Brouwer, A., El Khattabi, M., Poelmann, R.E., Gittenberger-de Groot, A.C., and Meijlink, F. (1995). Expression patterns of the paired-related homeobox genes MHox/Prx1 and S8/Prx2 suggest roles in development of the heart and the forebrain. Mech. Dev. *52*, 51–64.

Li, C., Alemany-Ribes, M., Raftery, R.M., Nwoko, U., Warman, M.L., and Craft, A.M. (2023). Directed differentiation of human pluripotent stem cells into articular cartilage reveals effects caused by absence of WISP3, the gene responsible for progressive pseudorheumatoid arthropathy of childhood. Ann. Rheum. Dis. *82*, 1547–1557.

Liu, H., Xu, J., Liu, C.-F., Lan, Y., Wylie, C., and Jiang, R. (2015). Whole transcriptome expression profiling of mouse limb tendon development by using RNA-seq. J. Orthop. Res. *33*, 840–848.

Loh, K.M., Chen, A., Koh, P.W., Deng, T.Z., Sinha, R., Tsai, J.M., Barkal, A.A., Shen, K.Y., Jain, R., Morganti, R.M., et al. (2016). Mapping the Pairwise Choices Leading from Pluripotency to Human Bone, Heart, and Other Mesoderm Cell Types. Cell *166*, 451–467.

Ma, N., Teng, X., Zheng, Q., and Chen, P. (2019). The regulatory mechanism of p38/MAPK in the chondrogenic differentiation from bone marrow mesenchymal stem cells. J. Orthop. Surg. *14*, 434–438.

Makris, E.A., Hu, J.C., and Athanasiou, K.A. (2013). Hypoxia-induced collagen crosslinking as a mechanism for enhancing mechanical properties of engineered articular cartilage. Osteoarthr. Cartil. *21*, 634–641.

Markman, S., Zada, M., David, E., Giladi, A., Amit, I., and Zelzer, E. (2023). A single-cell census of mouse limb development identifies complex spatiotemporal dynamics of skeleton formation. Dev. Cell *58*, 565–581.e4.

Martin, J.F., Bradley, A., and Olson, E.N. (1995). The paired-like homeo box gene MHox is required for early events of skeletogenesis in multiple lineages. Genes Dev. *9*, 1237–1249.

McInnes, L., Healy, J., Saul, N., and Großberger, L. (2018). UMAP: Uniform Manifold Approximation and Projection. J. Open Source Softw. *3*, 861.

Muddasani, P., Norman, J.C., Ellman, M., van Wijnen, A.J., and Im, H.-J. (2007). Basic fibroblast growth factor activates the MAPK and NFkappaB pathways that converge on Elk-1 to control production of matrix metalloproteinase-13 by human adult articular chondrocytes. J. Biol. Chem. *282*, 31409–31421.

Mundlos, S., Schwahn, B., Reichert, T., and Zabel, B. (1992). Distribution of osteonectin mRNA and protein during human embryonic and fetal development. J. Histochem. Cytochem. *40*, 283–291.

Muthuirulan, P., Zhao, D., Young, M., Richard, D., Liu, Z., Emami, A., Portilla, G., Hosseinzadeh, S., Cao, J., Maridas, D., et al. (2021). Joint disease-specificity at the regulatory base-pair level. Nat. Commun. *12*, 4161.

Niu, X., Melendez, D.L., Raj, S., Cai, J., Senadeera, D., Mandelbaum, J., Shestopalov, I.A., Martin, S.D., Zon, L.I., Schlaeger, T.M., et al. (2024). A conserved transcription factor regulatory program promotes tendon fate. Dev. Cell *59*, 3106–3123.e12.

Pacifici, M., Koyama, E., Shibukawa, Y., Wu, C., Tamamura, Y., Enomoto-Iwamoto, M., and Iwamoto, M. (2006). Cellular and Molecular Mechanisms of Synovial Joint and Articular Cartilage Formation. Ann. N. Y. Acad. Sci. *1068*, 74–86.

Pothiawala, A., Sahbazoglu, B.E., Ang, B.K., Matthias, N., Pei, G., Yan, Q., Davis, B.R., Huard, J., Zhao, Z., and Nakayama, N. (2022). GDF5+ chondroprogenitors derived from human pluripotent stem cells preferentially form permanent chondrocytes. Dev Camb Engl *149*, dev196220.

Raouf, A., Ganss, B., McMahon, C., Vary, C., Roughley, P.J., and Seth, A. (2002). Lumican is a major proteoglycan component of the bone matrix. Matrix Biol. *21*, 361–367.

Richard, D., Liu, Z., Cao, J., Kiapour, A.M., Willen, J., Yarlagadda, S., Jagoda, E., Kolachalama, V.B., Sieker, J.T., Chang, G.H., et al. (2020). Evolutionary Selection and Constraint on Human Knee Chondrocyte Regulation Impacts Osteoarthritis Risk. Cell *181*, 362–381.e28.

Richard, D., Pregizer, S., Venkatasubramanian, D., Raftery, R.M., Muthuirulan, P., Liu, Z., Capellini, T.D., and Craft, A.M. (2023). Lineage-specific differences and regulatory networks governing human chondrocyte development. eLife *12*, e79925.

Sakai, T., and Kumagai, K. (2025). Molecular dissection of tendon development and healing: Insights into tenogenic phenotypes and functions. J. Biol. Chem. *301*, 108353.

Satoh, A., Gardiner, D.M., Bryant, S.V., and Endo, T. (2007). Nerve-induced ectopic limb blastemas in the axolotl are equivalent to amputation-induced blastemas. Dev. Biol. *312*, 231–244.

Seo, H.-S., and Serra, R. (2007). Deletion of Tgfbr2 in Prx1-cre expressing mesenchyme results in defects in development of the long bones and joints. Dev. Biol. *310*, 304–316.

Serra, R., Johnson, M., Filvaroff, E.H., LaBorde, J., Sheehan, D.M., Derynck, R., and Moses, H.L. (1997). Expression of a Truncated, Kinase-Defective TGF-β Type II Receptor in Mouse Skeletal Tissue Promotes Terminal Chondrocyte Differentiation and Osteoarthritis. J. Cell Biol. *139*, 541–552.

Shwartz, Y., Viukov, S., Krief, S., and Zelzer, E. (2016). Joint Development Involves a Continuous Influx of Gdf5-Positive Cells. Cell Rep. *15*, 2577–2587.

Sim, Y.-J., Kim, M.-S., Nayfeh, A., Yun, Y.-J., Kim, S.-J., Park, K.-T., Kim, C.-H., and Kim, K.-S. (2017). 2i Maintains a Naive Ground State in ESCs through Two Distinct Epigenetic Mechanisms. Stem Cell Rep. *8*, 1312–1328.

Smith, C.A., Humphreys, P.A., Naven, M.A., Woods, S., Mancini, F.E., O'Flaherty, J., Meng, Q.-J., and Kimber, S.J. (2023). Directed differentiation of hPSCs through a simplified lateral plate mesoderm protocol for generation of articular cartilage progenitors. PLoS One *18*, e0280024.

Spagnoli, A., O'Rear, L., Chandler, R.L., Granero-Molto, F., Mortlock, D.P., Gorska, A.E., Weis, J.A., Longobardi, L., Chytil, A., Shimer, K., and Moses, H.L. (2007). TGF-β signaling is essential for joint morphogenesis. J. Cell Biol. *177*, 1105–1117.

Storm, E.E., and Kingsley, D.M. (1999). GDF5 coordinates bone and joint formation during digit development. Dev. Biol. *209*, 11–27.

Tani, S., Okada, H., Onodera, S., Chijimatsu, R., Seki, M., Suzuki, Y., Xin, X., Rowe, D.W., Saito, T., Tanaka, S., et al. (2023). Stem cell-based modeling and single-cell multiomics reveal gene-regulatory mechanisms underlying human skeletal development. Cell Rep. *42*, 112276.

Traag, V.A., Waltman, L., and van Eck, N.J. (2019). From Louvain to Leiden: guaranteeing well-connected communities. Sci. Rep. *9*, 5233.

Tsumaki, N., Tanaka, K., Arikawa-Hirasawa, E., Nakase, T., Kimura, T., Thomas, J.T., Ochi, T., Luyten, F.P., and Yamada, Y. (1999). Role of CDMP-1 in Skeletal Morphogenesis: Promotion of Mesenchymal Cell Recruitment and Chondrocyte Differentiation. J. Cell Biol. *144*, 161–173.

Tzchori, I., Day, T.F., Carolan, P.J., Zhao, Y., Wassif, C.A., Li, L., Lewandoski, M., Gorivodsky, M., Love, P.E., Porter, F.D., et al. (2009). LIM homeobox transcription factors integrate signaling events that control three-dimensional limb patterning and growth. Development *136*, 1375–1385.

Untergasser, G., Gander, R., Lilg, C., Lepperdinger, G., Plas, E., and Berger, P. (2005). Profiling molecular targets of TGF-beta1 in prostate fibroblast-to-myofibroblast transdifferentiation. Mech. Ageing Dev. *126*, 59–69.

Wang, Q., Green, R.P., Zhao, G., and Ornitz, D.M. (2001). Differential regulation of endochondral bone growth and joint development by FGFR1 and FGFR3 tyrosine kinase domains. Development *128*, 3867–3876.

Wu, C.L., Dicks, A., Steward, N., Tang, R., Katz, D.B., Choi, Y.R., and Guilak, F. (2021). Single cell transcriptomic analysis of human pluripotent stem cell cho ndrogenesis. Nat. Commun. *12*, 362.

Ying, Q.L., Nichols, J., Chambers, I., and Smith, A. (2003). BMP induction of Id proteins suppresses differentiation and sustains embryonic stem cell self-renewal in collaboration with STAT3. Cell *115*, 281–292.

Ying, S., Shiraishi, A., Kao, C.W., Converse, R.L., Funderburgh, J.L., Swiergiel, J., Roth, M.R., Conrad, G.W., and Kao, W.W. (1997). Characterization and expression of the mouse lumican gene. J. Biol. Chem. *272*, 30306–30313.

Zhang, C.-H., Gao, Y., Jadhav, U., Hung, H.-H., Holton, K.M., Grodzinsky, A.J., Shivdasani, R.A., and Lassar, A.B. (2021). Creb5 establishes the competence for Prg4 expression in articular cartilage. Commun. Biol. *4*, 332.

Stem Cell Reports, Volume *20*

Supplemental Information

The roles of TGF-β, Wnt, and MAPK signaling pathways in joint lineage specification *in vitro* and *ex vivo*

Suyash Raj, Thomas Cutia, Stefano Menghini, Mireia Alemany-Ribes, Junming Cai, Mariel Young, Sarah K. Jachim, Terence D. Capellini, and April M. Craft

The roles of TGFβ, Wnt, and MAPK signaling pathways in joint lineage specification *in vitro* and *ex vivo*

Suyash Raj[1], Thomas Cutia[1], Stefano Menghini[1], Mireia Alemany-Ribes[1,2], Junming Cai[1], Mariel Young[3], Sarah K. Jachim[1], Terence D. Capellini[3,4], April M. Craft[1,2,5]

Affiliations

[1] Department of Orthopedic Surgery, Boston Children's Hospital, Boston, MA, United States

[2] Department of Orthopedic Surgery, Harvard Medical School, Boston, MA, United States

[3] Human Evolutionary Biology, Harvard University, Cambridge, MA, United States

[4] Broad Institute of MIT and Harvard, Cambridge, MA, United States

[5] Harvard Stem Cell Institute, Cambridge, MA, United States

*corresponding author: April.Craft@childrens.harvard.edu

Supplementary information

Supplemental Figures S1–S6

Supplemental Experimental Procedures

Table S1. Metadata and differentially expressed genes between raw clusters in individual Seurat objects, related to Figure S4.

Table S2. Differentially expressed genes between named clusters in the integrated Seurat object, related to Figure 4 and Figure S5.

Table S3. Differentially expressed genes between the Chondrogenic cluster of TGFβ and TGFβ+IWP2 and differentially expressed genes between the Connective Tissue 2 cluster of T+PD and T+βFGF, related to Figure 5.

Supplemental Figure 1

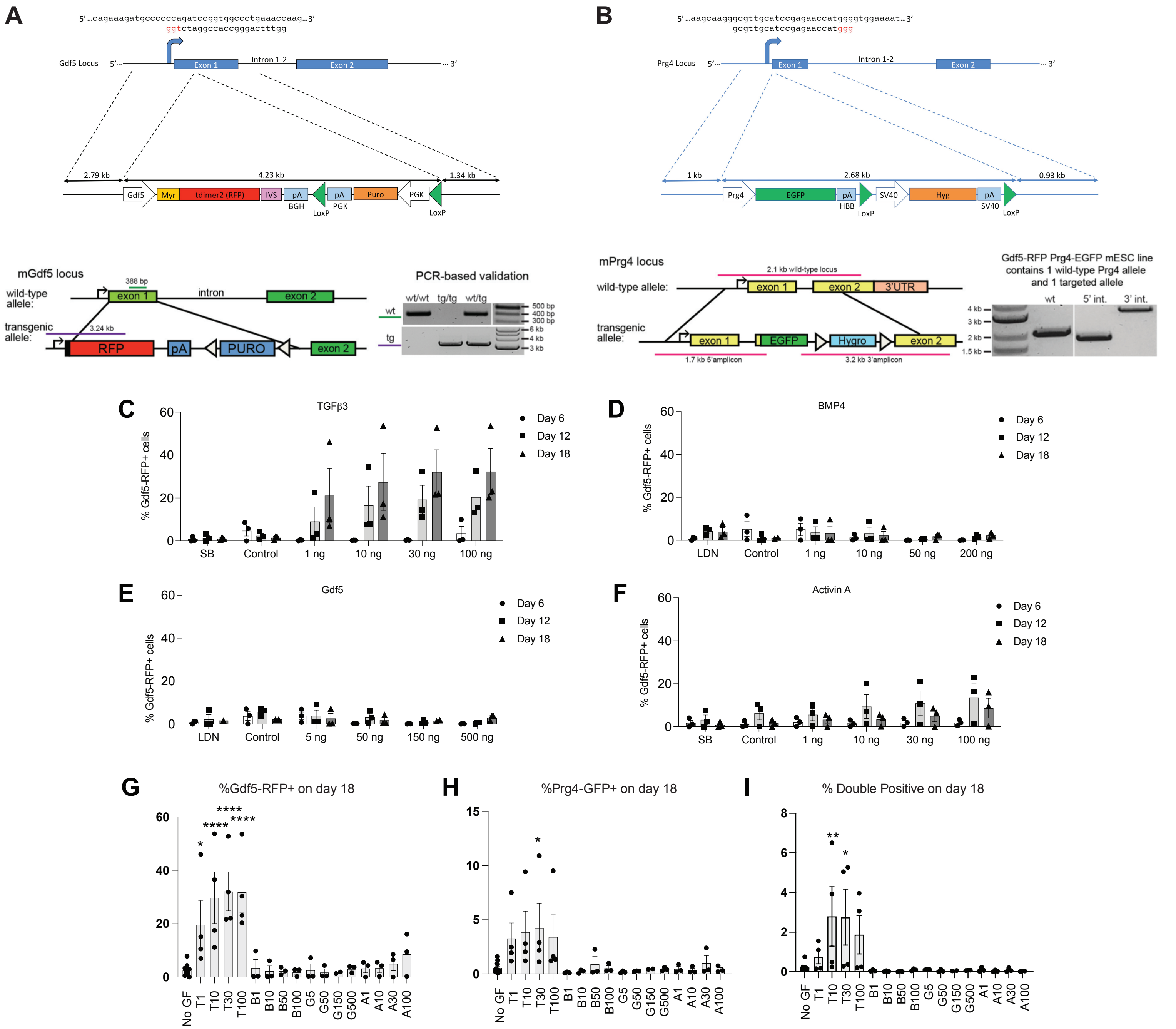

Figure S1. CRISPR-Cas9-mediated generation of the BC29 dual reporter mESC line and activation of the reporters by TGFβ signaling pathway ligands.

(A) Gene targeting and homologous recombination strategy to insert transgene into the Gdf5-RFP locus (top), accompanied by PCR confirmation of recombination (bottom). PCR products were confirmed by sequencing.

(B) Gene targeting and homologous recombination strategy to insert transgene into the Prg4-GFP locus (top), accompanied by PCR confirmation of recombination (bottom). PCR products were confirmed by sequencing.

(C) Percent of Gdf5-RFP expressing cells on days 6, 12 and 18 of micromass culture in the presence or absence (control) of increasing concentrations of TGFβ3 or its antagonist SB431542 (SB), quantified by flow cytometry. Icons represent independent experiments.

(D) Percent of Gdf5-RFP expressing cells on days 6, 12 and 18 of micromass culture in the presence or absence of increasing concentrations of BMP4 or its antagonist LDN-193189 (LDN). Icons represent independent experiments.

(E) Percent of Gdf5-RFP expressing cells on days 6, 12 and 18 of micromass culture in the presence or absence of increasing concentrations of Gdf5 or its antagonist LDN-193189. Icons represent independent experiments.

(F) Percent of Gdf5-RFP expressing cells on days 6, 12 and 18 of micromass culture in the presence or absence of increasing concentrations of Activin A or its antagonist SB431542. Icons represent independent experiments.

(G) Percent of Gdf5-RFP expressing cells on day 18 of micromass culture in the presence or absence of increasing concentrations (numerical values in ng/ml) of TGFβ3 (T), BMP4 (B), Gdf5 (G) or Activin A (A), or no growth factors (No GF). Icons represent independent experiments. Values, mean +/- SEM. ANOVA with Dunnett's multiple comparison correction, *$p<0.05$, **$p<0.01$, ***$p<0.001$, ****$p<0.0001$ versus no GF.

(H) Percent of Prg4-GFP expressing cells on day 18 of micromass culture in the presence or absence of increasing concentrations (numerical values in ng/ml) of TGFβ3 (T), BMP4 (B), Gdf5 (G) or Activin A (A), or no growth factors (No GF). Icons represent independent experiments. Values, mean +/- SEM. ANOVA with Dunnett's multiple comparison correction, *$p<0.05$, **$p<0.01$, ***$p<0.001$, ****$p<0.0001$ versus no GF.

(I) Percent of double positive cells on day 18 of micromass culture in the presence or absence of increasing concentrations (numerical values in ng/ml) of TGFβ3 (T), BMP4 (B), Gdf5 (G) or Activin A (A), or no growth factors (No GF). Icons represent independent experiments. Values, mean +/- SEM. ANOVA with Dunnett's multiple comparison correction, *$p<0.05$, **$p<0.01$, ***$p<0.001$, ****$p<0.0001$ versus no GF.

Supplemental Figure 2

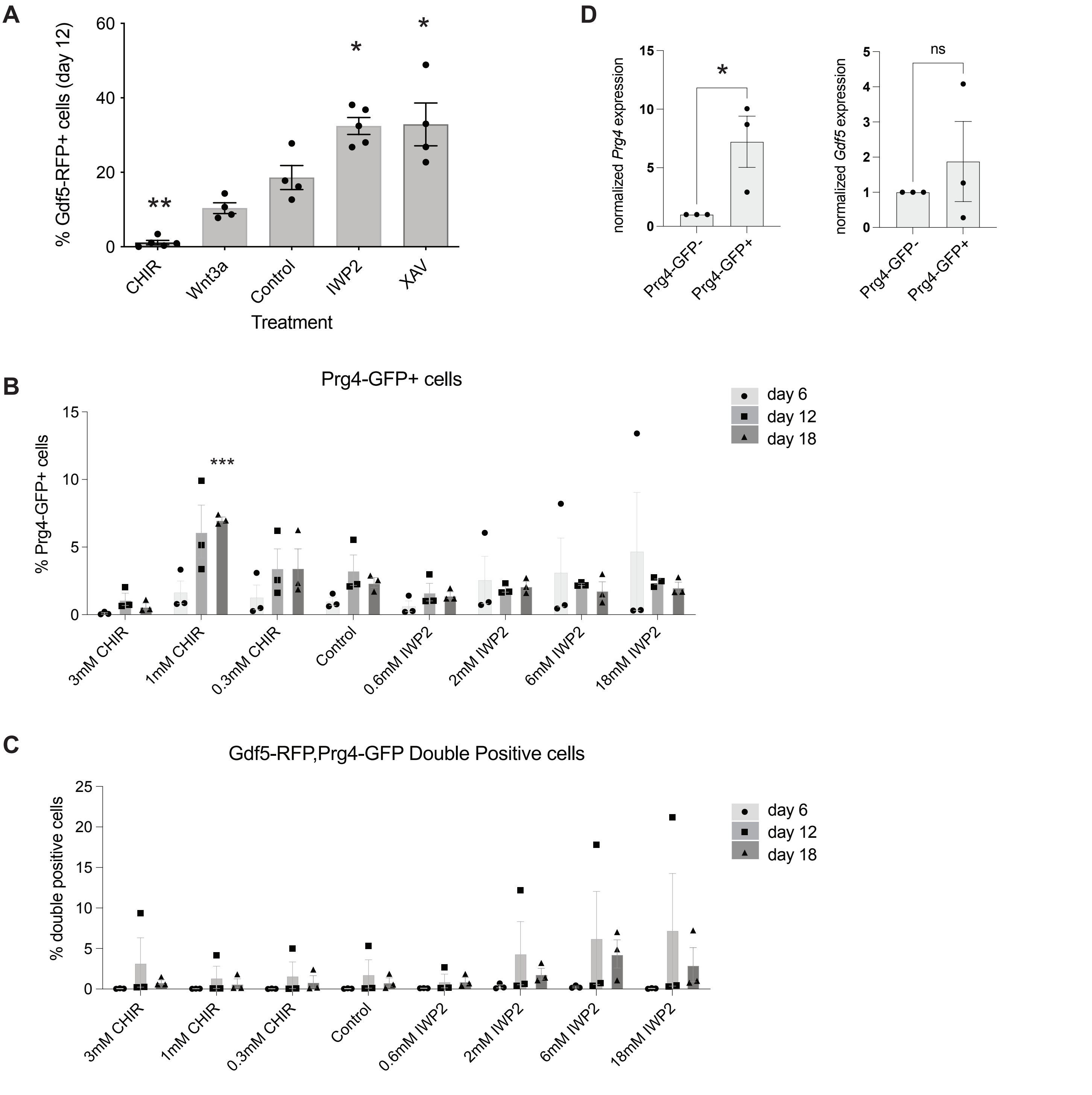

Figure S2. Expression of Gdf5-RFP and Prg4-GFP following modulation of Wnt.
(A) Percentage of Gdf5-RFP expressing cells on day 12 of micromass culture following indicated treatment. n=4-5 independent experiments. Values, mean +/- SEM. One-way ANOVA with Dunnett's multiple comparison correction, *p<0.05, **p<0.01, versus control (TGFβ3 alone). XAV, XAV939 (2 μM), Wnt3a (100 ng/mL).
(B) Percentage of Prg4-GFP expressing cells on days 6, 12, and 18 of micromass culture in the presence or absence of increasing concentrations of the agonist CHIR99021 or the antagonist IWP2. All treatments include TGFβ3 (10 ng/mL). n=3 independent experiments. Values, mean +/- SEM. One-way ANOVA with Dunnett's multiple comparison correction, ***p<0.001, versus control within each timepoint (TGFβ3 alone).
(C) Percentage of Gdf5-RFP;Prg4-GFP (double positive) expressing cells on days 6, 12, and 18 of micromass culture in the presence or absence of increasing concentrations of the agonist CHIR99021 or the antagonist IWP2. n=3 independent experiments, values, mean +/- SEM. One-way ANOVA with Dunnett's multiple comparison correction, all treatments not significant versus control within each timepoint.
(D) Normalized expression of *Prg4* and *Gdf5* mRNA following cell sorting of Prg4-GFP+ and Prg4-GFP+ cells from 3 independent experiments (cultured in the presence of TGFβ3). n=3 independent experiments. Values, mean +/- SEM, Student's t-test, *p<0.05.

Supplemental Figure 3

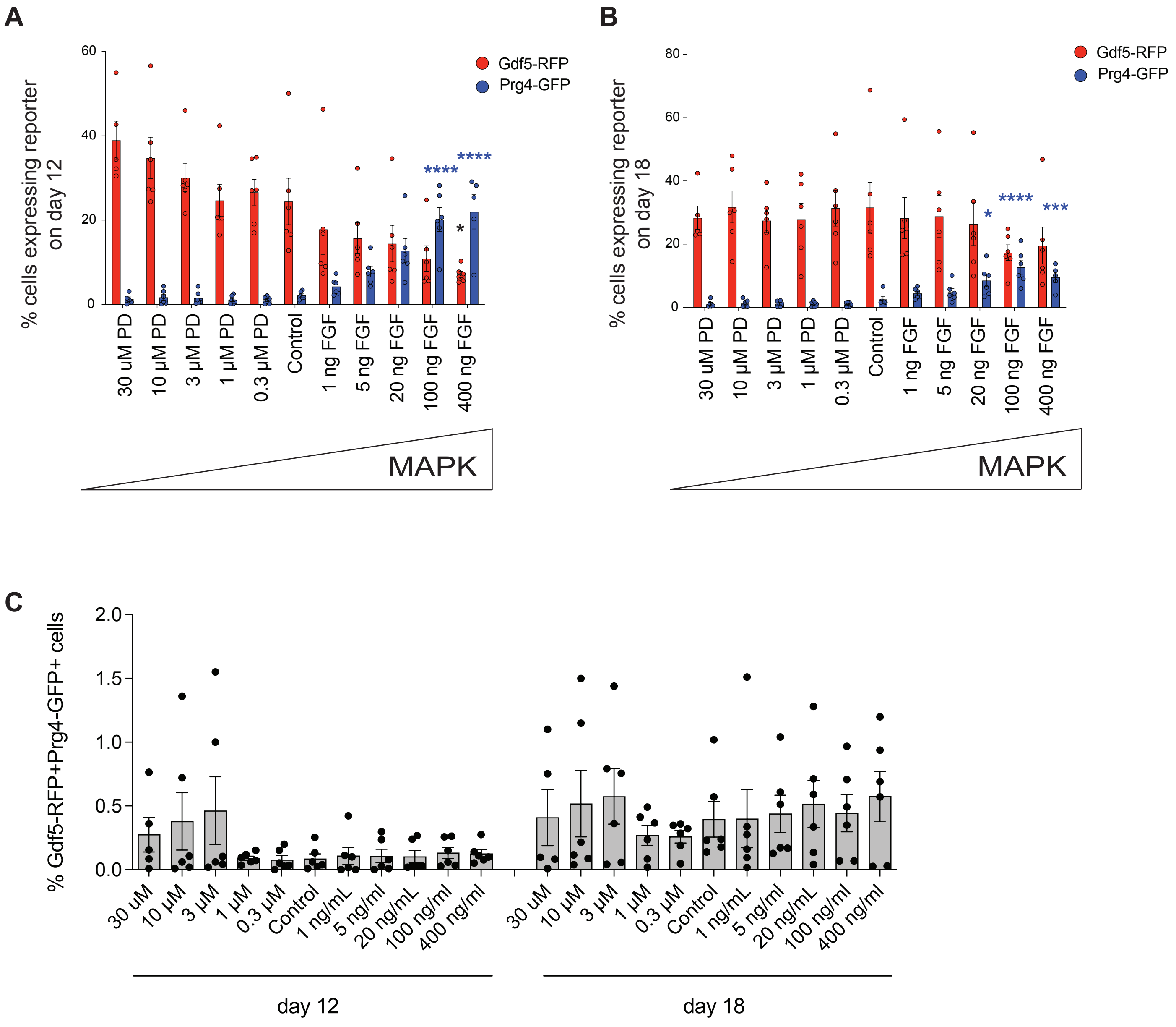

Figure S3. Flow cytometric quantification of Gdf5-RFP and Prg4-GFP following modulation of MAPK signaling.
(A) Percentage of Gdf5-RFP (red bars) or Prg4-GFP (blue bars) expressing cells on day 12 of micromass culture following indicated treatment. Icons represent independent experiments. Values, mean +/- SEM. ANOVA with Dunnett's multiple comparison correction, *p< 0.05, **p< 0.01, ***p<0.001, ****p<0.0001 versus control (TGFβ3 alone).
(B) Percentage of Gdf5-RFP (red bars) or Prg4-GFP (green bars) expressing cells on day 18 of micromass culture following indicated treatment. Icons represent independent experiments. Values, mean +/- SEM. ANOVA with Dunnett's multiple comparison correction, *p< 0.05, **p< 0.01, ***p<0.001, ****p<0.0001 versus control (TGFβ3 alone).
(C) Percentage of Gdf5-RFP;Prg4-GFP-expressing cells (double positive) on day 12 and 18 of micromass culture. Icons represent independent experiments. Values, mean+/- SEM. ANOVA with Dunnett's multiple comparison correction versus control (TGFβ3 alone), results are not significant.

Supplemental Figure 4

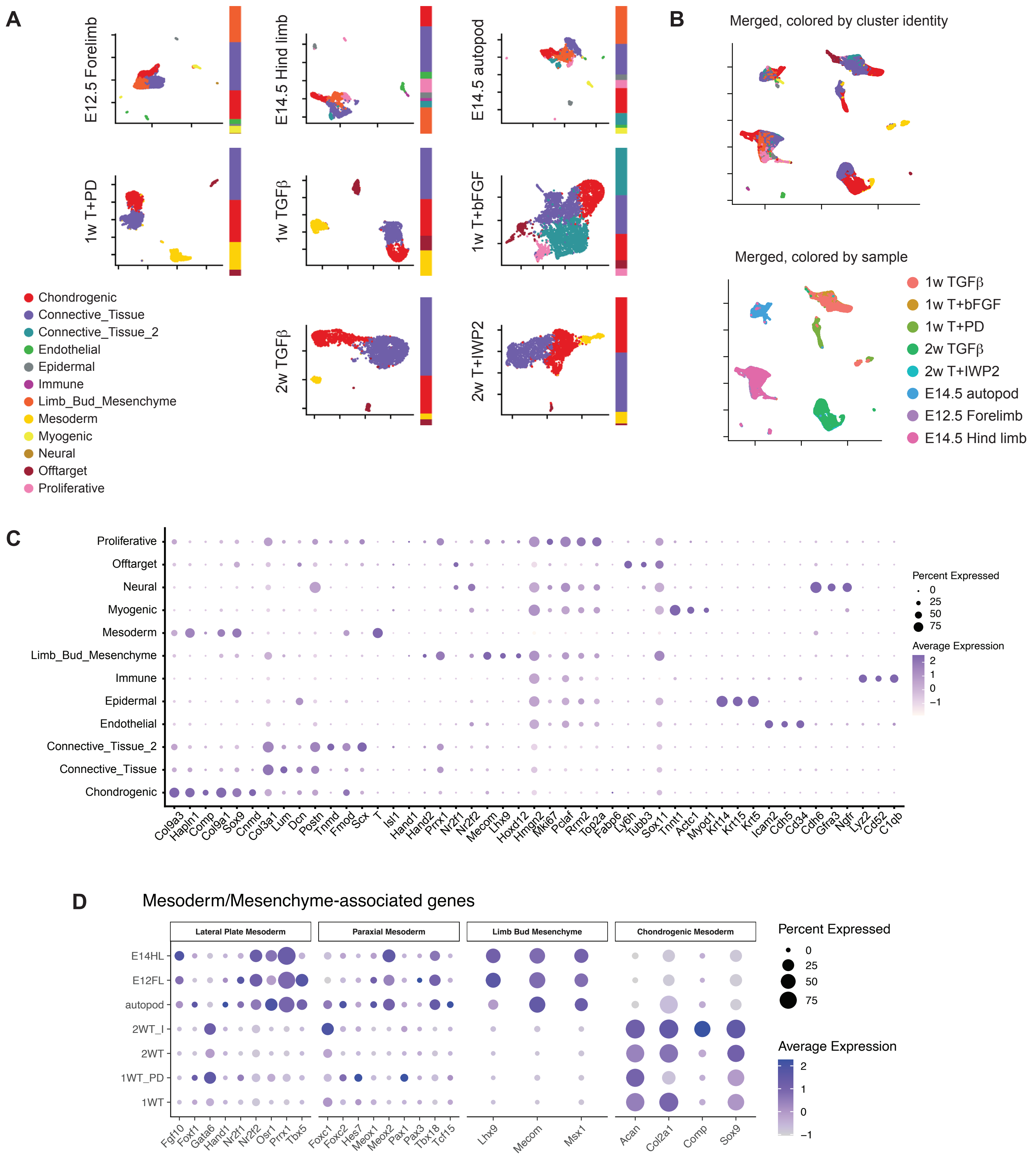

Figure S4. UMAPs and cluster identification of *in vitro* and *in vivo* samples.
(A) UMAPs with cluster identification and proportions in each sample.
(B) Merged RNA object with cluster identification (left) and colored by sample (right).
(C) Semi-quantitative dotplot showing percentage of cells and normalized level of gene expression in each cluster in the merged RNA object (B).
(D) Dotplot showing percentage of cells and normalized level of gene expression in each sample's respective Mesoderm cluster (*in vitro*) or Limb_Bud_Mesenchyme cluster (*in vivo*). Mesoderm clusters (*in vitro*) and Limb_Bud_Mesenchyme clusters (*in vivo*) from the merged RNA object were subset to assess the levels of mesoderm/mesenchyme-associated genes.

Supplemental Figure 5

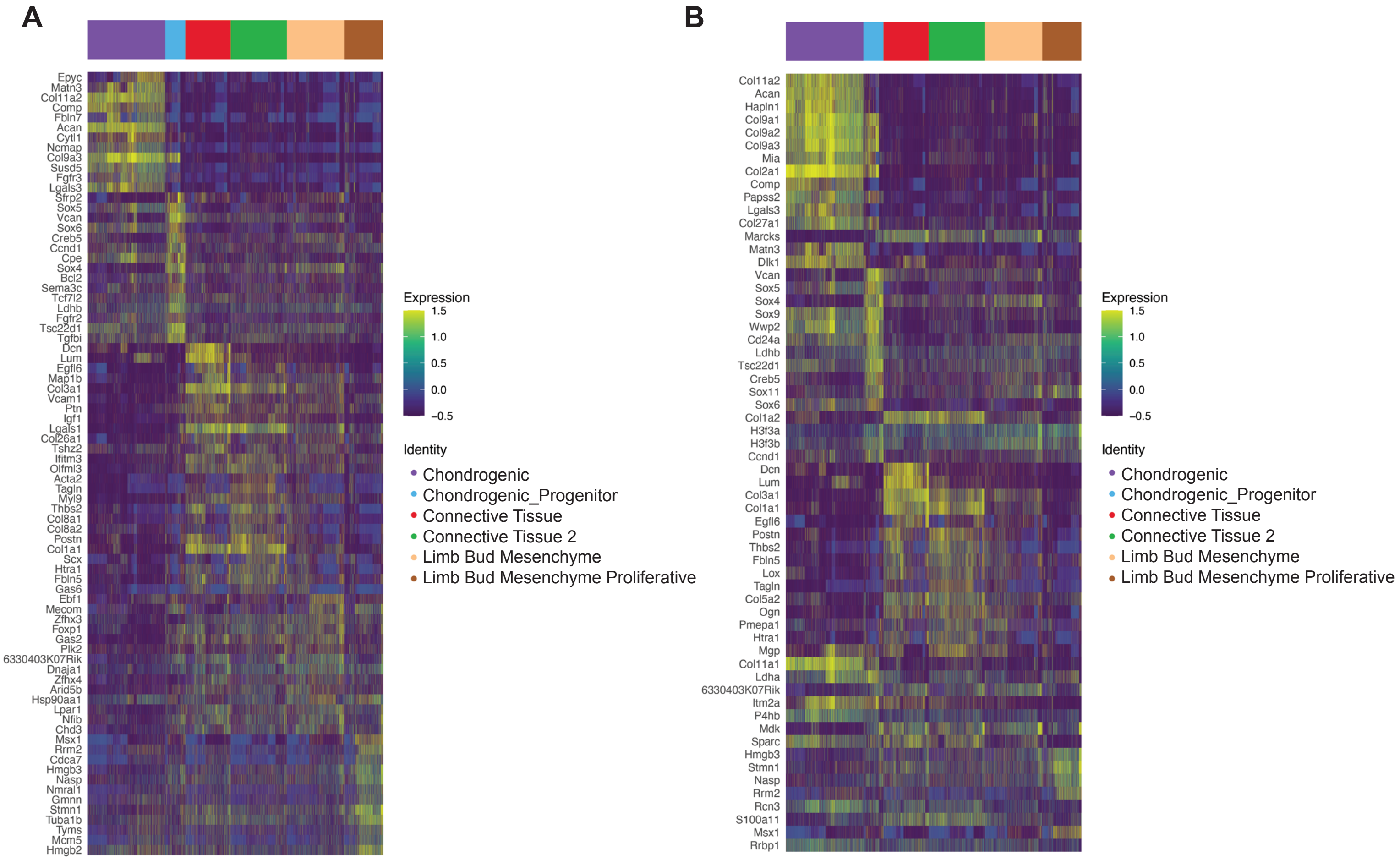

Figure S5. DEGs in integrated object clusters.
(A) Top DEGs per cluster by log(2)FC.
(B) Top DEGs per cluster by p-adj value.

Supplemental Figure 6

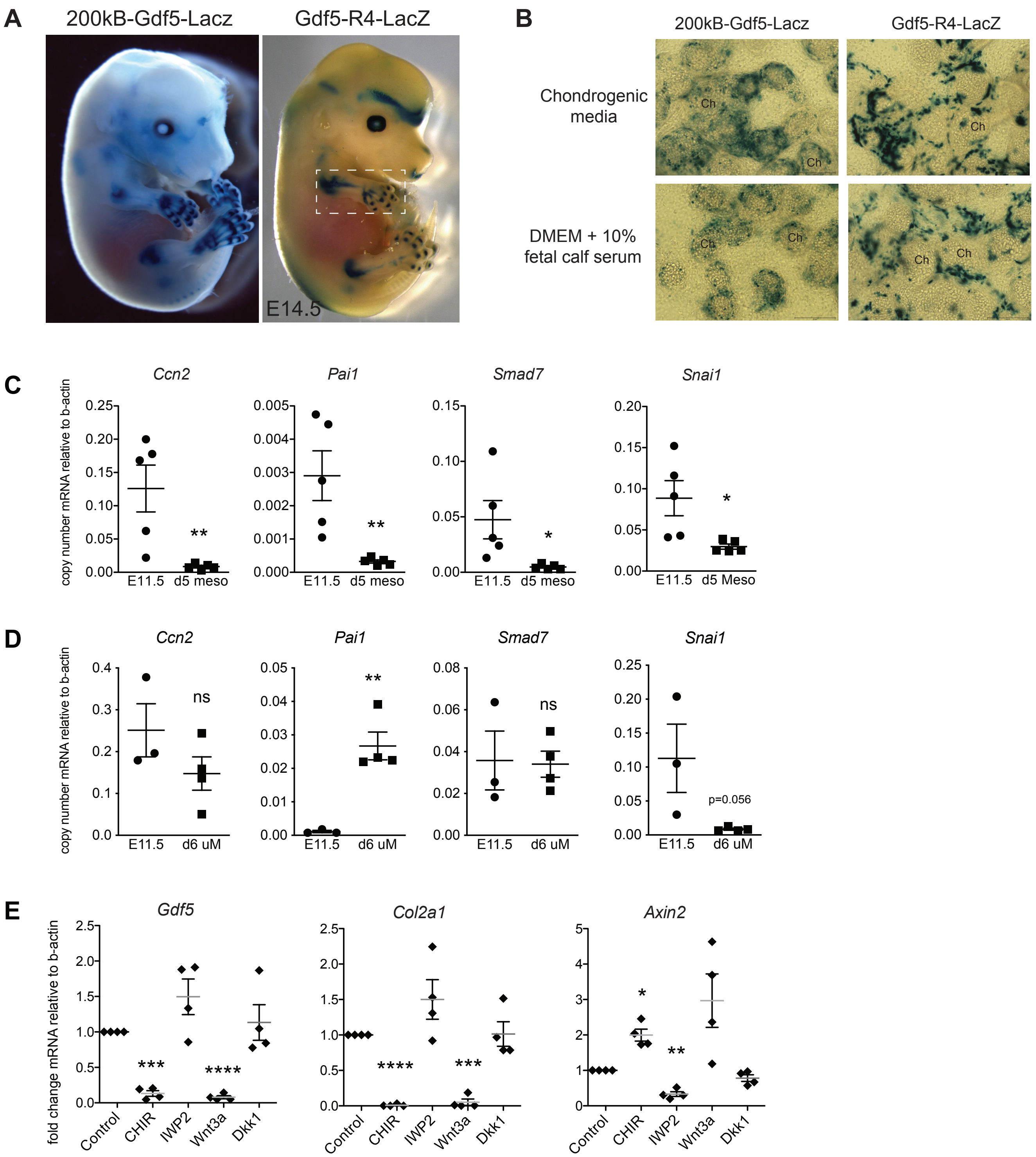

Figure S6. Characterization of mouse embryonic limb bud micromass cultures

(A) X-gal staining of representative mouse embryos on embryonic day 14.5 (E14.5) shows a restricted joint-specific pattern of LacZ-expressing cells in the Gdf5-R4-LacZ transgenic mouse (PHC21) compared to the 200kB-Gdf5-LacZ transgenic mouse, which has a broader expression domain including joint cells and chondrogenic cells.

(B) Localization of LacZ-expressing cells following 8 days of micromass culture in chondrogenic media used in mESC micromass culture (top panels) or traditional limb bud micromass media DMEM + 10% fetal calf serum (bottom panels). Cells from E11.5 limb buds were isolated and plated in micromass culture for 8 days in indicated media. Ch, chondrogenic node. Scale bar, 200 μm.

(C) RT-qPCR of genes associated with TGFβ signaling in pooled limb buds from FVB strain embryos at E11.5 and mESC-derived mesoderm on day 5 of differentiation. n = 5 independent experiments, Values, mean +/- SEM. Student's t-test, *p<0.05, **p<0.01.

(D) RT-qPCR of genes associated with TGFβ signaling in pooled limb buds from FVB strain embryos at E11.5 (n = 3 independent experiments) and mESC-derived cells on day 6 of micromass culture in the presence of TGFβ3 (n = 4 independent experiments). Values, mean +/- SEM. Student's t-test, **p<0.01, ns, not significant.

(E) RT-qPCR quantified expression of *Gdf5, Col2a1* and *Axin2* in 4 day old micromass cultures derived from E11.5 FVB strain mouse embryonic limb buds following treatment with recombinant proteins that modulate canonical Wnt signaling compared to small molecule agonist and antagonist treatments. n=4 independent experiments. ANOVA with Dunnett's multiple comparison correction, *p<0.05, **p<0.01, ***p<0.001, ****p<0.0001 vs control.

Supplemental Experimental Procedures

Generation of dual mESC reporter line

The E14 mouse cell line was modified for this study. This is a male embryonic stem cell line that was initially generated from mouse strain 12910la. The coding sequence for myristoylated tdRFP was introduced into the Gdf5 locus and eGFP was introduced into the Prg4 locus of E14 mESCs using CRISPR-Cas9 targeting and homologous recombination (Cong *et al*, 2013) to generate the BC29 dual reporter mESC line (Fig. S1A). The Gdf5-RFP targeting plasmid contained tdRFP and a LoxP-flanked puromycin cassette within a 5' arm homologous to the proximal promoter region and a 3' arm homologous to exon 2 and the 3'UTR. A linear piece of DNA containing the transgenic construct within the 5' homologous region and the 3' homology region was nucleofected into E14 mESCs along with the PX330 plasmid (Addgene #42230) containing a guide RNA. Puromycin resistant clones were screened for homologous recombination by PCR and confirmed by Sanger sequencing. The puromycin resistance cassette was removed by transfection of a Cre-recombinase expression plasmid, selection of puromycin sensitive clones, and the lack of puromycin coding sequence was confirmed in the BC29 clone by Sanger sequencing. The reporter gene eGFP and a hygromycin selection cassette were introduced into the Prg4 locus using CRISPR-Cas9 using homologous recombination and confirmed by Sanger sequencing (Fig. S1B). We note that the untargeted allele of Prg4 is missing one cysteine codon in the signal peptide. The pX330-U6-Chimeric_BB-CBh-hSpCas9 plasmid was a gift from Feng Zhang (Addgene plasmid # 42230; RRID:Addgene_42230).

ESC maintenance and differentiation

Parental E14 and BC29 mESCs were maintained in a modified serum-free (SF), feeder-free culture system as described previously (Craft *et al*, 2013; Gadue *et al*, 2006; Ying *et al*, 2003). Serum-free media was supplemented GSK3β inhibitor CHIR99021 and the MEK inhibitor PD0325901 (i.e., 2i media) (Sim *et al*, 2017). For differentiation, ESCs were dissociated and cultured in suspension in serum-free differentiation medium (SFD) (Gouon-Evans *et al*, 2006) without additional growth factors for 48 hours. Embryoid bodies (EBs) were then dissociated and re-aggregated in SFD with the addition of growth factors or inhibitors as indicated [9 ng/ml activin A (inhibin, beta A), 1 µM CHIR99021, 150 ng/ml noggin or 0.1 µM LDN-193189]. EBs were harvested 28 hours later, the cells were dissociated and re-aggregated at 250,000 cells/ml in 24-well ULA dishes (Costar) or 5% polyheme (Poly 2-Hydroxyethyl methacrylate) (Millipore Sigma) coated 6-well plates for 48 hours in SFD containing 10 ng/ml bFGF (FGF2) and 10 µM Y-27632. Aggregates were harvested on day 5 and dissociated to single-cell suspension.

Three dimensional micromass cultures were generated from mESC-derived mesoderm cells on day 5. Briefly, 250,000 cells were seeded onto Matrigel (Corning)-coated wells of 24-well tissue culture plates (Falcon) in 20 µl of chondrogenic media consisting of high glucose DMEM with 1x ITS supplement (Thermofisher), ascorbic acid (50 µg/ml, Sigma-Aldrich), proline (40 µg/ml, Sigma-Aldrich) and dexamethasone (0.1 µM, Sigma-Aldrich). Chondrogenic media was added to cover the adherent cells after 1 h. Recombinant growth factors and small molecules were purchased from R&D Systems and Sigma-Aldrich.

The day of micromass plating was noted as day 0. Micromass cultures were treated on day 2 and day 4 of micromass culture with indicated factors:

Factor	Concentrations
BMP4	1 – 200 ng/mL
Activin A	1 – 200 ng/mL
Gdf5	1 – 200 ng/mL
TGFβ3	1 – 100 ng/mL
IWP2	0.6 – 18 μM
CHIR99021	0.3 – 3 μM
FGF2 (bFGF	1 – 400 ng/mL
PD0325901	0.3 – 30 μM
SB431542	5.4 μM
LDN-193189	0.1 μM

On day 6, cultures were either (i) maintained in their respective treatments or (ii) cultured in chondrogenic media supplemented with TGFβ3 (10 ng/ml) alone, with media changes every 2-3 days. Micromass cultures were harvested as indicated, primarily at days 6, 12, and 18 of micromass culture for gene expression and flow cytometric analyses. Cells/tissues were maintained in a 5% CO_2 environment for the duration of the culture period.

Mouse embryonic limb bud cultures

Animal studies were performed in compliance of ethical regulations and were approved by Animal Resources at Children's Hospital or Harvard University. Wild type FVB mice, transgenic 200kb BAC Gdf5 LacZ mice, and *PHC21* (*Gdf5-R4-LacZ*) mice (both FVB strain) were used for these studies. Limb buds were micro-dissected from mouse E11.5, E12.5, and E14.5 stage embryos. Cells were isolated from limb buds using 0.2% type I collagenase for up to two hours at 37°C. Cells were then plated in micromass culture as described above or subjected to single cell transcriptomic analyses as described below.

Flow cytometry and cell sorting

EBs generated from mESC differentiation experiments were dissociated by incubation with TrypLE (Invitrogen) and stained with following antibodies: anti-mouse Flk-1-biotin, anti-mouse Pdgfrα (CD140a)-allophycocyanin (APC; clone APA5, eBioscience, San Diego, CA, USA), streptavidin-phycoerythrin (PE) or streptavidin-PE-Cy7 (BD Pharmingen). Antibody stains were performed at 4°C in PBS containing 5% (v/v) fetal calf serum (FCS). Micromass cultures derived from mESCs, embryonic limb buds, and limb bud micromass cultures were dissociated using 0.2% type I collagenase. Limb bud-derived cells expressing beta-galactosidase (*Gdf5-R4-LacZ* transgene) were detected using the fluorescein di-V-galactoside (FDG) reagent within the FluoReporter LacZ flow cytometry kit (F1930, Thermofisher) following manufacturer's instructions. Cells were analyzed using a BD FACS Fortessa (Becton Dickinson). Cell sorting was performed using a BD Melody (Becton Dickinson). Dead

cells staining positively with Draq7 (D15105, ThermoFisher) were excluded from analyses. Analysis was performed using FlowJo (Tree Star). Mean, standard errors and all statistical tests were calculated in Prism (Graphpad).

Reverse Transcription Quantitative PCR (RT-qPCR) Quantitative real-time PCR

Total RNA was extracted using the MagMAX mirVana Total RNA kit (Applied Biosystems). RNA (0.1-1 µg) was reverse transcribed with Superscript IV VILO reverse transcriptase (Invitrogen) and treated with ezDNase enzyme (Invitrogen). RT-qPCR was performed on a ViiA 7 Real-Time PCR System with OptiFlex Optics System (Applied Biosystems) using PowerUp SYBR Green PCR kit (Applied Biosystems). Genomic DNA standards were used to evaluate the efficiency of the PCR and calculate the copy number of each gene relative to the expression of *β-actin*. Primers used are listed in the table below. Biological replicates/independent experiments are indicated in figure and figure legends. Mean, standard errors and all statistical tests were calculated in Prism (Graphpad).

Primers used for quantitative real-time PCR

GENE	Forward (5'-3')	Reverse (5'-3')
Gdf5	ACCACGCAGTCATTCAGACCCTAA	TGTTGGCAGAGTCGATGAAGAGGA
Prg4	AGCCAATGAAGAAGTGCACAGGGA	AGGTGTGTGTCTGGAAAGGTCCAA
Col2a1	CCAAACACTTTCCAACCGCAGTCA	AGTCTGCCCAGTTCAGGTCTCTTA
Snai1	GTCTCAGAAGGGACCATGAATAA	ATAGTTCTGGGAGACACATTGG
Ccn2 (Ctgf)	CAAATGCTGTGCAGGTGATAAA	CCTGAGCCAGCCATTTCTTA
Pai1	GGGCACAACACTTTCATTCAG	ACAGTGGACCTTGAGATAGGA
Scx	AAGTTGAGCAAAGACCGTGAC	AGTGGCATCCACCTTCACTA
Axin2	AAGAGAGACCAAGCCGATTGCTGA	AGTCACTAACACGGCGCTACTCAT

Histology, staining and imaging

Micromass cultures derived from limb bud cells were fixed in 4% paraformaldehyde prior to standard X-gal and alcian blue staining protocols. Images were taken using an EVOS Auto FL 2.0 microscope. *In vitro*-derived micromass tissues were cryofrozen in Tissue-Tek optimal cutting temperature (OCT) mounting medium (Sakura, 4583). 5-10 µm sections of micromass tissues derived from mESCs were cut, fixed in 4% paraformaldehyde, and counterstained with DAPI-containing mounting media. Adjacent tissue sections were fixed in 4% paraformaldehyde and stained with Toluidine blue (Electron Microscopy Sciences) to visualize proteoglycans. Live mESC-derived cultures and fluorescent microscopy was performed using a Zeiss LCM800 confocal microscope.

10x chromium scRNA-seq Library Preparation

In vivo and *in vitro* samples were dissociated using collagenase and resuspended at a concentration of one million cells per mL. 10,000 cells from each sample were loaded into the 10x Genomics Chromium Controller.

Libraries were then prepared using Single Cell 3' Library & Gel Bead Kit v2 (10X Genomics). Final cDNA quality was analyzed using a Bioanalyzer (Agilent). Sequencing was performed by the Harvard Medical School Biopolymers Facility using an Illumina NextSeq 500 to approximately 30,000 reads per cell.

scRNA-seq Processing

Sequencing reads were processed by Cell Ranger (version 3.0.0, 10X Genomics). Reads were aligned to the GRCm38(version 100) genome with added RFP and EGFP transgenes. After genome annotation and filtering of empty droplets, 3000 cells at minimum were captured for each sample. The gene barcode matrix was then passed onto Velocyto (version 0.17.0) which produced individual barcode matrices for spliced, unspliced, and ambiguous mRNA (La Manno *et al*, 2018).

Samples were input into Seurat (version 5.2.1). Cells that had less than 500 UMIs or more than 70,000 UMIs were removed. Low quality cells that contained more than 20% mitochondrial DNA were removed from the final dataset. The cell matrix was normalized using SCTransform (Hafemeister & Satija, 2019) which uses Pearson residuals from a regularized negative binomial regression in order to normalize and scale the matrix as well as to regress out the effects driven by cell cycle stages, mitochondrial genes, and technical variation.

Unsupervised clustering and Projection

Uniform Manifold Approximation and Projection (UMAP) was used to perform dimensional reduction and visualization of the clusters (McInnes *et al*, 2018). The python implementation of UMAP-learn was specifically used. The distance metric for UMAP used was Pearson's correlation, which has been shown to outperform Euclidean distance and does not require minimum principal components (Kim *et al*, 2019). Clustering was performed using Leiden algorithm (Traag *et al*, 2019).. The appropriate resolution was identified using the Clustree package (Zappia & Oshlack, 2018). Clusters were identified through Differentially Expressed Genes (DEGs) and gene ontology. The MAST framework was used to identify DEGs for each cluster (Finak *et al*, 2015).

Integration Analysis

To compare *in vitro* and *in vivo* samples, shared cell types were integrated using reciprocal PCA (RPCA). Seurat includes an implementation of RPCA which was used for our analysis (Butler *et al*, 2018). Mouse E14.5 hind limb and E14 hind limb autopod were used as the reference for the integration because they contained the most diverse populations. Feature plots and dot plots were generated using the scaled RNA matrix, and the volcano plot was generated using the enhanced volcano (version 1.24.0) package.

Computational packages and versions used in these analyses

Package	Version	Package	Version
cowplot	1.1.3	MatrixGenerics	1.18.1
EnhancedVolcano	1.24.0	matrixStats	1.5.0
ggrepel	0.9.6	patchwork	1.3.0
enrichR	3.4	SeuratDisk	0.0.0.9021
extrafont	0.19	RColorBrewer	1.1-3
limma	3.62.2	viridis	0.6.5
harmony	1.2.3	viridisLite	0.4.2
Rcpp	1.0.14	writexl	1.5.2
future.apply	1.11.3	readxl	1.4.5
future	1.34.0	lubridate	1.9.4
glmGamPoi	1.18.0	forcats	1.0.0
RcppAnnoy	0.0.22	stringr	1.5.1
plotly	4.10.4	purrr	1.0.4
scales	1.3.0	readr	2.1.5
MAST	1.32.0	tidyr	1.3.1
SingleCellExperiment	1.28.1	tibble	3.2.1
SummarizedExperiment	1.36.0	ggplot2	3.5.1
Biobase	2.66.0	tidyverse	2.0.0
GenomicRanges	1.58.0	dplyr	1.1.4
GenomeInfoDb	1.42.3	reticulate	1.42.0
IRanges	2.40.1	Seurat	5.2.1
S4Vectors	0.44.0	SeuratObject	5.0.2
BiocGenerics	0.52.0	sp	2.2-0
